# Patient Outcomes After Long-Term Acute Care Hospital Closures

**DOI:** 10.1001/jamanetworkopen.2023.44377

**Published:** 2023-11-21

**Authors:** Anica C. Law, Nicholas A. Bosch, Yang Song, Archana Tale, Robert W. Yeh, Jeremy M. Kahn, Jennifer P. Stevens, Allan J. Walkey

**Affiliations:** 1 The Pulmonary Center, Department of Medicine, Boston University School of Medicine, Boston, Massachusetts; 2 Richard A and Susan F Smith Center for Outcomes Research, Beth Israel Deaconess Medical Center, Boston, Massachusetts; 3 Department of Critical Care Medicine, University of Pittsburgh, Pittsburgh, Pennsylvania; 4 Center for Healthcare Delivery Science, Beth Israel Deaconess Medical Center, Boston, Massachusetts; 5 Division of Pulmonary, Critical Care, and Sleep Medicine, Department of Medicine, Beth Israel Deaconess Medical Center, Boston, Massachusetts; 6 Evans Center for Implementation and Improvement Sciences, Boston University School of Medicine, Boston, Massachusetts; 7 Department of Health Law, Policy and Management, Boston University School of Public Health, Boston, Massachusetts

## Abstract

**Importance:**

Long-term acute care hospitals (LTCHs) are common sites of postacute care for patients recovering from severe respiratory failure requiring mechanical ventilation (MV). However, federal payment reform led to the closure of many LTCHs in the US, and it is unclear how closure of LTCHs may have affected upstream care patterns at short-stay hospitals and overall patient outcomes.

**Objective:**

To estimate the association between LTCH closures and short-stay hospital care patterns and patient outcomes.

**Design, Setting, and Participants:**

This retrospective, national, matched cohort study used difference-in-differences analysis to compare outcomes at short-stay hospitals reliant on LTCHs that closed during 2012 to 2018 with outcomes at control hospitals. Data were obtained from the Medicare Provider Analysis and Review File, 2011 to 2019. Participants included Medicare fee-for-service beneficiaries aged 66 years and older receiving MV for at least 96 hours in an intensive care unit (ie, patients at-risk for prolonged MV) and the subgroup also receiving a tracheostomy (ie, receiving prolonged MV). Data were analyzed from October 2022 to June 2023.

**Exposure:**

Admission to closure-affected hospitals, defined as those discharging at least 60% of patients receiving a tracheostomy to LTCHs that subsequently closed, vs control hospitals.

**Main Outcomes and Measures:**

Upstream hospital care pattern outcomes were short-stay hospital do-not-resuscitate orders, palliative care delivery, tracheostomy placement, and discharge disposition. Patient outcomes included hospital length of stay, days alive and institution free within 90 days, spending per days alive within 90 days, and 90-day mortality.

**Results:**

Between 2011 and 2019, 99 454 patients receiving MV for at least 96 hours at 1261 hospitals were discharged to 459 LTCHs; 84 LTCHs closed. Difference-in-differences analysis included 8404 patients (mean age, 76.2 [7.2] years; 4419 [52.6%] men) admitted to 45 closure-affected hospitals and 45 matched-control hospitals. LTCH closure was associated with decreased LTCH transfer rates (difference, −5.1 [95% CI −8.2 to −2.0] percentage points) and decreased spending-per-days-alive (difference, −$8701.58 [95% CI, −$13 323.56 to −$4079.60]). In the subgroup of patients receiving a tracheostomy, there was additionally an increase in do-not-resuscitate rates (difference, 10.3 [95% CI, 4.2 to 16.3] percentage points) and transfer to skilled nursing facilities (difference, 10.0 [95% CI, 4.2 to 15.8] percentage points). There was no significant association of closure with 90-day mortality.

**Conclusions and Relevance:**

In this cohort study, LTCH closure was associated with changes in discharge patterns in patients receiving mechanical ventilation for at least 96 hours and advanced directive decisions in the subgroup receiving a tracheostomy, without change in mortality. Further studies are needed to understand how LTCH availability may be associated with other important outcomes, including functional outcomes and patient and family satisfaction.

## Key Points

**Question:**

Were federal payment reform and the subsequent closure of long-term acute care hospitals (LTCHs) associated with upstream hospital care practices and outcomes for patients receiving mechanical ventilation?

**Findings:**

In this cohort study of 8404 Medicare beneficiaries receiving mechanical ventilation for at least 96 hours at 45 closure-affected hospitals and 45 matched controls, LTCH closure was associated with decreased LTCH transfer and decreased spending; in the subgroup of patients receiving a tracheostomy, LTCH closure was additionally associated with increased use of do-not-resuscitate orders and transfer to skilled nursing facilities; there was no change in mortality.

**Meaning:**

These findings suggest that discharge patterns and advanced directive decisions were sensitive to availability of postacute care options of mechanically ventilated patients.

## Teaser

This cohort study estimates the association of long-term acute care hospital closures with short-stay hospital care patterns and patient outcomes.

## Introduction

In the US, patients who survive critical illness but have longer-term, complex medical needs may remain in the acute, short-stay hospitals to which they were initially admitted, be transferred to a skilled nursing facility (SNF), or be transferred a long-term acute care hospital (LTCH) for postacute care.^1,2,3^ Like short-stay hospitals, LTCHs aim to provide advanced interdisciplinary care (eg, ventilator weaning by pulmonologists and respiratory therapists) and often have higher nurse-to-patient ratios than SNFs.^4,5^ LTCHs were created in the 1980s as an administrative exemption from the acute care hospital Prospective Payment System for 40 chronic disease hospitals and are currently defined by patients’ mean length of stay (LOS; ie, ≥25 days). Because of longer LOS compared with short-stay hospitals, the Centers for Medicare & Medicaid Services (CMS) historically reimbursed LTCHs at higher rates, leading to rapid LTCH growth in the early 2000s.^6,7^ However, since 2005, CMS implemented a series of reforms designed to restrict growth and spending.^6^ Most recently, the Pathway for Sustainable Growth Rate Reform Act of 2013 reserved higher reimbursement rates for care of patients requiring more complex care, such as those receiving prolonged mechanical ventilation (MV)^6^; reimbursement for patients not meeting complexity requirements was gradually reduced to hospital-level reimbursement rates.^8^ Due to resulting financial pressures, many LTCHs closed,^8^ curtailing one option for postacute care for some short-stay hospitals. How CMS payment reform and subsequent LTCH closures might affect upstream hospital care patterns (eg, tracheostomy placement, palliative care, do-not-resuscitate orders) and overall outcomes (eg, spending and mortality) among patients at risk for prolonged MV is unclear.

The use of LTCHs for postacute care of hospitalized patients varies widely, depending on regional LTCH availability and transfer practices at individual hospitals.^9,10^ To estimate the association of LTCH closures with upstream short-stay hospital care patterns and patient outcomes, we leveraged hospital variation in use of closing LTCHs and conducted a difference-in-differences study among patients at risk for or receiving prolonged MV. As prior work has shown that earlier discharges to LTCHs (and by extension, earlier tracheostomy) are cost-saving for hospitals (which receive a single prospective payment regardless of LOS),^1^ we hypothesized that LTCH closure would be associated with changes in upstream care patterns (ie, decreases in tracheostomy rates, increases in do-not-resuscitate orders, and shifts in discharge patterns) but not with patient outcomes.

## Methods

### Patient Cohort

This cohort study was deemed exempt from review and informed consent by Beth Israel Deaconess Medical Center and Boston University Institutional Review Boards because it was non–human participants research. This study is reported following the Strengthening the Reporting of Observational Studies in Epidemiology (STROBE) reporting guideline for observational studies. We used data from the Medicare Provider Analysis and Review File (MedPAR) and the Master Beneficiary Summary File from 2011 to 2019, which contain demographic and clinical data on hospital, LTCH, and SNF admissions. We linked to the CMS Chronic Conditions Warehouse^11^ to obtain detailed comorbidity data from prior health care encounters. Additional hospital characteristics were obtained from the 2016 American Hospital Association annual survey database.

We identified Medicare fee-for-service beneficiaries aged 66 years and older who were hospitalized between 2011 to 2019 with admission to an intensive care unit (ICU; per revenue center codes). Patients receiving prolonged MV, defined as those receiving MV for at least 96 hours and tracheostomy,^12,13^ are known to have complex postacute care needs.^7,14^ We identified patients at risk for prolonged MV (ie, receipt of MV ≥96 hours; *International Classification of Diseases, Ninth Revision* [*ICD-9*] procedure code 96.72 or *International Statistical Classification of Diseases and Related Health Problems, Tenth Revision* [*ICD-10*] 5A1955Z,^15,16^ who are known to have a high rate of progression to prolonged mechanical ventilation^15,16^) and the subgroup receiving prolonged MV (ie, additional receipt of tracheostomy; *ICD-9* codes 31.1 and 31.23; *ICD-10* codes 0B11[0–4]F4 and 0B11[0–4]Z4).^12,13^ Evaluating patients receiving MV for 96 hours or longer allowed evaluation of associations between LTCH closure and treatment decisions made during acute respiratory failure (eg, tracheostomy placement).

LTCHs were defined by CMS Health Cost Reporting Information System reports (2011-2019).^5,17^ LTCHs that ceased operation between 2012 and 2018 were defined as closing LTCHs. We defined LTCH transfers as temporally adjacent hospital and LTCH admissions, where the hospital admission ended on date *n* and the LTCH admission began on date *n* or *n* + 1, a method that has been validated over the use of the discharge destination field in MedPAR data.^17^

### Exposure

Difference-in-differences analyses compare dichotomous exposures, but hospitals vary on a continuum in the proportions of patients transferred to LTCHs and the numbers of different LTCHs used as transfer destinations. Our prior work showed that hospitals in the US transfer approximately 60% of patients receiving tracheostomies to LTCHs for postacute care.^3^ Therefore, to estimate outcomes associated with near-total loss of LTCH availability, we defined closure-affected hospitals as hospitals discharging at least 60% of patients receiving a tracheostomy to a closing LTCH in the year prior to closure, ie, hospitals that had relied on closing LTCHs for most or all postacute care of patients receiving a tracheostomy (a group with unchanged eligibility for higher CMS reimbursement rates). The exposure was defined at the hospital level because the use of LTCHs for postacute care is often dependent on institutional partnerships between hospitals and LTCHs and ecologic exposures can reduce patient-level confounding.^18^

### Outcomes

We examined 2 groups of outcomes. Upstream outcomes occurring during the acute short-stay hospitalization included do-not-resuscitate orders (*ICD-9* code V49.86; *ICD-10* code Z66), palliative care delivery (*ICD-9* code V66.7; *ICD-10* code Z51.5), tracheostomy rates, and disposition at the end of hospitalization (ie, LTCH transfer, SNF transfer, home discharge, facility or home hospice transfer, and in-hospital mortality). Patient outcomes included hospital LOS, alive and institution-free days within 90 days of admission (days not in a hospital, LTCH, or SNF^3,15^), spending per days alive within 90 days of admission (sum of Medicare, out-of-pocket, and other payer payments,^19^ divided by total days alive within 90 days of admission), and mortality within 90 days of admission. All outcomes, except for tracheostomy rates, were assessed for patients receiving MV for 96 hours or longer and the subgroup who received a tracheostomy.

### Statistical Analysis

**Figure 1.**
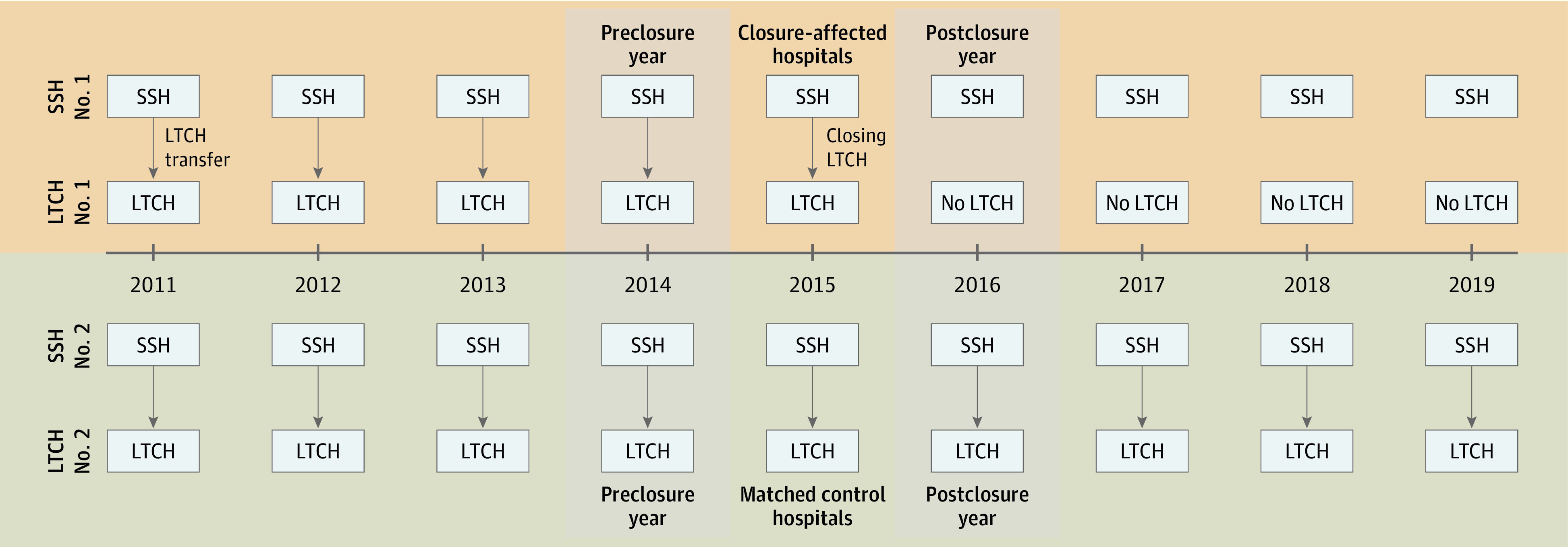
Study Design The years before and after long-term acute care hospitals (LTCH) closure were identified as preclosure and postclosure years. In difference-in-difference analysis, control short-stay hospitals (SSHs) were matched to closure-affected SSHs by hospital characteristics; control hospitals contributed the same years of data for analysis as their matched closure-affected SSH.

Difference-in-differences analyses can achieve unbiased effect estimates, even when intervention and control cohorts have differences in underlying characteristics. Still, we minimized possible confounding by 1:1 matching closure-affected hospitals to control, non–closure-affected hospitals (ie, hospitals that had not discharged any patients receiving a tracheostomy to a closing LTCH) on hospital characteristics^20^: teaching hospital status, ownership (for profit, private nonprofit, or public), safety-net hospital status (per CMS Impact File: hospitals with a Medicare Disproportional Share Index in the top quartile per US region), region (Northeast, Midwest, South, and West), the number of beds per hospital, and the proportion of patients receiving tracheostomies who were discharged to LTCHs in the year prior to exposure year. After exposure and control hospital pairs were identified, the 1 year before and after closure of an LTCH were used as the pre-exposure and postexposure years for exposed hospitals while excluding the year of LTCH closure; matched control hospitals each contributed the same years of data (Figure 1).

After matching hospitals, difference-in-differences analyses were performed at the patient level using multivariable hierarchical regression with 200-fold bootstrapping, adjusting for hospital- and patient-level characteristics to further reduce confounding between exposure and patient outcome. Model variables included difference-in-differences variables estimating the association of LTCH closure with patient outcomes (absolute change)^21^; hospital- and patient-level covariables as fixed effects (year and hospital characteristics plus rural vs urban status; patient sex, age, race and ethnicity, Social Vulnerability Index,^22^ Medicare and Medicaid dual eligibility, surgical status,^23^ chronic comorbidities^11^ [30 CMS Chronic Conditions Warehouse comorbidities, with multiyear preadmission lookback per CMS algorithms], and acute severity of illness^24^ [acute cardiovascular, neurologic, hematologic, liver, or kidney dysfunction; validated against Sequential Organ Failure Assessment score^24^]); and matched pairs of closure-affected and control hospitals of admission as a random effect to account for within-hospital and matched-hospital clustering. We chose not to adjust for local market factors, including distance to LTCH or number of LTCHs in hospital referral regions, which are known to be associated with discharge to LTCH but not directly with patient outcomes (ie, these are sometimes used as instruments in instrumental variable analysis^25^) and thereby could not serve as confounders. Patient race and ethnicity (categorized as non-Hispanic Black, non-Hispanic White, and other [eg, Asian or Pacific Islander, Hispanic, and all other groups]) were determined from the Medicare Master Beneficiary Summary File, which derives self-reported race and ethnicity from the Social Security Administration. We included race and ethnicity in fully adjusted models because these have been associated with differences in end-of-life care practices and outcomes among Medicare beneficiaries. Less than 1% of patients had missing data (Social Vulnerability Index); these patients were dropped in adjusted analysis.

To assess the validity of difference-in-difference analyses, we conducted 2 falsification tests using outcomes unlikely to be related to LTCH availability but associated with severity of illness: incidence of septic shock (*ICD-9* codes 995.92 and 785.52; *ICD-10* codes R6520 and R6521) and number of chronic comorbidities. As an alternative method to account for unobserved heterogeneity in hospital characteristics, we conducted a sensitivity analysis using hospital of admission as a fixed effect rather than random effect. We also conducted 2 sensitivity analyses assessing lower exposure strengths (eg, hospitals that may have alternate local LTCHs); we varied the definition of closure-affected hospitals to those discharging at least 30% or discharging more than 0% of patients receiving a tracheostomy to a closing LTCH in the year prior to closure. Finally, because standard 2-way fixed-effect difference-in-difference analyses involving staggered treatment timing can lead to bias,^26^ we additionally estimated difference-in-differences using the Callaway and Sant’Anna method for staggered difference-in-differences,^26^ which reduces bias through the estimation of group-time average treatment effects (ie, separate average treatment effects for each time period of treatment, which are then aggregated by dynamic time using a weighted mean approach). While aggregating by dynamic time produces average treatment effects for varying lengths of time after exposure (in this case, 1-7 years after LTCH closure), our estimate of interest was average treatment effect 1 year after LTCH closure to parallel our primary analysis. Staggered difference-in-differences models were adjusted for the same fixed (patient and hospital characteristics) and random (matched hospital clustering) effects as our primary static difference-in-difference models. Statistical testing was 2-tailed, with α = .05. Analyses were conducted using SAS version 9.4 (SAS Institute). Data were analyzed from October 2022 to June 2023.

## Results

Between 2011 and 2019, 584 973 eligible patients receiving MV for at least 96 hours were admitted to 1287 hospitals and 99 454 patients admitted to 1261 hospitals were transferred to 459 LTCHS. Between 2012 and 2018, 84 LTCHs closed, which had received patients from 219 hospitals. Of these, 46 hospitals (3.6%) had discharged at least 60% of patients receiving a tracheostomy to a closing LTCH in the year prior to LTCH closure and were defined as closure-affected hospitals; 1031 hospitals (81.7%) had not discharged any patients to closing LTCHs; we matched 45 closure-affected hospitals to 45 control hospitals. eTable 1 in Supplement 1 shows characteristics of closure-affected and control hospitals before and after selection of hospital matches, and eTable 2 in Supplement 1 shows characteristics of the patients admitted to these hospitals between 2011 and 2019 before and after selection of hospital matches. After restricting to patients admitted in the year before and after an LTCH closure, a total of 8404 patients (mean [SD] age, 76.2 [7.2] years; 4419 [52.6%] men) were included in difference-in-difference analysis; patient-level baseline characteristics at closure-affected and control hospitals are shown in Table 1, and characteristics of the tracheostomy subgroup are shown in eTable 3 in Supplement 1.

**Table 1.** Characteristics of Included Patients

Characteristics	Patients, No. (%)			
	Preclosure period		Postclosure period	
	Closure-affected hospital (n = 2170 patients at 45 hospitals)	Matched control hospital (n = 2112 patients at 45 hospitals)	Closure-affected hospital (n = 2005 patients at 45 hospitals)	Matched control hospital (n = 2117 patients at 45 hospitals)
Age, mean (SD), y	76.6 (7.3)	76.0 (7.2)	76.2 (7.3)	76.1 (7.1)
Sex				
Male	1100 (50.7)	1123 (53.2)	1001 (49.9)	1195 (56.4)
Female	1070 (49.3)	989 (46.8)	1004 (50.1)	922 (43.6)
Race and ethnicity				
Black, non-Hispanic	438 (20.2)	228 (10.8)	435 (21.7)	225 (10.6)
White, non-Hispanic	1643 (75.7)	1770 (83.8)	1486 (74.1)	1786 (84.4)
Other^a^	89 (4.1)	114 (5.4)	84 (4.2)	106 (5.0)
Social vulnerability index, mean (SD)^b^	0.5 (0.3)	0.5 (0.3)	0.5 (0.3)	0.5 (0.3)
Medicaid dual eligibility	476 (21.9)	440 (20.8)	398 (19.9)	421 (19.9)
Surgical patient	970 (44.7)	869 (41.1)	856 (42.7)	919 (43.4)
Comorbidities				
Alzheimer disease or dementia	789 (36.4)	650 (30.8)	725 (36.2)	679 (32.1)
Atrial fibrillation	811 (37.4)	779 (36.9)	740 (36.9)	808 (38.2)
Cancer	416 (19.2)	391 (18.5)	375 (18.7)	393 (18.6)
Congestive heart failure	1577 (72.7)	1404 (66.5)	1397 (69.7)	1430 (67.5)
Chronic kidney disease	1704 (78.5)	1624 (76.9)	1590 (79.3)	1722 (81.3)
Chronic obstructive pulmonary disease and bronchiectasis	1207 (55.6)	1064 (50.4)	1052 (52.5)	1078 (50.9)
Depressive disorders	765 (35.3)	732 (34.7)	732 (36.5)	784 (37.0)
Diabetes	1195 (55.1)	1108 (52.5)	1126 (56.2)	1158 (54.7)
Hip or pelvic fracture	71 (3.3)	90 (4.3)	68 (3.4)	82 (3.9)
Hyperlipidemia	1516 (69.9)	1423 (67.4)	1423 (71.0)	1497 (70.7)
Hypertension	2023 (93.2)	1930 (91.4)	1867 (93.1)	1980 (93.5)
Ischemic heart disease	1588 (73.2)	1461 (69.2)	1389 (69.3)	1499 (70.8)
Stroke or transient ischemic attack	581 (26.8)	549 (26.0)	564 (28.1)	547 (25.8)
Acute organ dysfunction				
Cardiovascular	1259 (58.0)	1139 (53.9)	1103 (55.0)	1204 (56.9)
Neurologic	785 (36.2)	811 (38.4)	825 (41.1)	921 (43.5)
Hematologic	529 (24.4)	489 (23.2)	438 (21.8)	523 (24.7)
Hepatic	143 (6.6)	148 (7.0)	161 (8.0)	173 (8.2)
Kidney	1324 (61.0)	1247 (59.0)	1192 (59.5)	1327 (62.7)
Hospital size				
Small (1-200 beds)	244 (11.2)	272 (12.9)	209 (10.4)	246 (11.6)
Medium (201-400 beds)	843 (38.8)	676 (32.0)	742 (37.0)	627 (29.6)
Large (>400 beds)	1083 (49.9)	1164 (55.1)	1054 (52.6)	1244 (58.8)
Ownership				
For profit	203 (9.4)	212 (10.0)	160 (8.0)	163 (7.7)
Private nonprofit	1811 (83.5)	1691 (80.1)	1723 (85.9)	1783 (84.2)
Public	156 (7.2)	209 (9.9)	122 (6.1)	171 (8.1)
Teaching hospital	1938 (89.3)	1828 (86.6)	1782 (88.9)	1859 (87.8)
Safety net hospital	639 (29.4)	480 (22.7)	574 (28.6)	530 (25.0)
Hospital region				
Northeast	112 (5.2)	241 (11.4)	113 (5.6)	205 (9.7)
Midwest	232 (10.7)	322 (15.2)	250 (12.5)	387 (18.3)
South	1727 (79.6)	1460 (69.1)	1515 (75.6)	1435 (67.8)
West	99 (4.6)	89 (4.2)	127 (6.3)	90 (4.3)
Prior LTCH use, median (IQR)^c^	0.61 (0.27-0.80)	0.71 (0.46-0.82)	0.60 (0.27-0.80)	0.71 (0.50-0.82)
Rural	27 (1.2)	43 (2.0)	23 (1.1)	54 (2.6)
Year of admission				
2011	471 (21.7)	341 (16.1)	0	0
2012	306 (14.1)	224 (10.6)	0	0
2013	193 (8.9)	184 (8.7)	481 (24.0)	329 (15.5)
2014	38 (1.8)	46 (2.2)	215 (10.7)	200 (9.4)
2015	78 (3.6)	87 (4.1)	183 (9.1)	220 (10.4)
2016	406 (18.7)	380 (18.0)	53 (2.6)	36 (1.7)
2017	678 (31.2)	850 (40.2)	79 (3.9)	100 (4.7)
2018	0	0	340 (17.0)	378 (17.9)
2019	0	0	654 (32.6)	854 (40.3)

Abbreviation: LTCH, long-term acute care hospital.

^a^ Includes Asian or Pacific Islander, Hispanic, and all other groups.

^b^ Social Vulnerability Index for each patient’s census tract is based on 15 social factors, including poverty, lack of vehicle access, and crowded housing. A census tract’s index is a percentile ranking and reflects the proportion of tracts in the country that are equal to or lower in terms of social vulnerability.

^c^ Prior LTCH use indicates the proportion of patients receiving tracheostomy discharged to any LTCH in the year prior to LTCH closure.

Unadjusted proportions of outcomes among patients receiving MV for at least 96 hours and patients receiving a tracheostomy at closure-affected and matched control hospitals are shown in Table 2 and eTable 4 in Supplement 1. Between preclosure and postclosure years, at both closure-affected and control hospitals, unadjusted rates of tracheostomy decreased among patients receiving MV for at least 96 hours; palliative care provision and do-not-resuscitate orders rates increased in patients receiving MV for at least 96 hours (Table 2) and the subgroup receiving a tracheostomy (eTable 4 in Supplement 1).

**Table 2.** Unadjusted Outcomes of Patients Receiving MV for at Least 96 Hours at Closure-Affected and Matched Control Hospitals

Outcome	Events, No. (%)			
	Preclosure period		Postclosure -period	
	Closure-affected hospital (n = 2170)	Control hospital (n = 2112)	Closure-affected hospital (n = 2005)	Control hospital (n = 2117)
90-d Mortality	1350 (62.2)	1273 (60.3)	1245 (62.1)	1302 (61.5)
Tracheostomy	413 (19.0)	405 (19.2)	341 (17.0)	408 (19.3)
Palliative care delivery	448 (20.6)	544 (25.8)	539 (26.9)	615 (29.1)
DNR	509 (23.5)	518 (24.5)	551 (27.5)	621 (29.3)
IFD in 90 d				
Mean (SD)	12.3 (22.7)	13.8 (24.0)	14.7 (24.2)	15.0 (24.5)
Median (IQR)	0.0 (0.0-11.0)	0.0 (0.0-17.0)	0.0 (0.0-24.0)	0.0 (0.0-26.0)
Spending/d alive in 90 d, $				
Mean (SD)	88 029.76 (73 006.91)	76 556.62 (62 639.99)	83 553.68 (62 918.86)	85 673.35 (72 069.56)
Median (IQR)	62 119.16 (37 962.00-118 770.00)	53 542.61 (34 154.39-100 097.99)	60 886.29 (38 406.00-111 488.12)	59 071.00 (37 119.00-112 142.99)
Index hospital length of stay, d				
Mean (SD)	17.8 (12.3)	17.4 (11.8)	17.2 (10.8)	18.0 (14.0)
Median (IQR)	15.0 (10.0-22.0)	15.0 (10.0-22.0)	15.0 (10.0-22.0)	15.0 (10.0-22.0)
Hospital disposition, No. (%)				
LTCH	411 (18.9)	400 (18.9)	256 (12.8)	414 (19.6)
SNF or IRF	410 (18.9)	425 (20.1)	431 (21.5)	423 (20.0)
Hospice (facility or home)	260 (12.0)	226 (10.7)	230 (11.5)	254 (12.0)
Home	120 (5.5)	135 (6.4)	122 (6.1)	106 (5.0)
Death	817 (37.6)	814 (38.5)	815 (40.6)	845 (39.9)

Abbreviations: DNR, do not resuscitate; IFD, institution-free days; IRF, inpatient rehabilitation facility; LTCH, long-term acute care hospital; MV, mechanical ventilation; SNF, skilled nursing facility.

**Figure 2.**
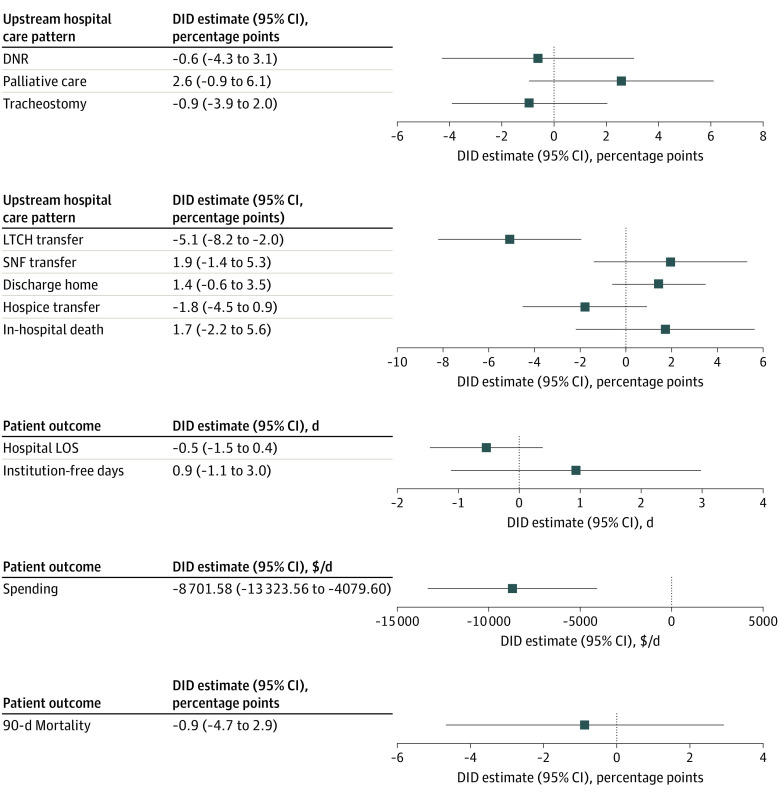
Change in Hospital Care Patterns and Patient Outcomes Associated With LTCH Closure at Closure-Affected Hospitals Compared With Matched Control Hospitals Among Patients Receiving Mechanical Ventilation for at Least 96 Hours Estimates and their 95% CIs of absolute change in outcomes were generated by difference-in-differences (DID) analysis adjusted for hospital- and patient-level characteristics, with matched exposure-control hospital pairs as random effect. DNR indicates do not resuscitate; LOS, length of stay; LTCH, long-term acute care hospital; SNF, skilled nursing facility.

In difference-in-differences analysis among patients receiving MV for at least 96 hours, LTCH closure was associated with decreased LTCH transfer (difference, −5.1 [95% CI, −8.2 to −2.0] percentage points) and decreased spending per days alive within 90 days of admission (difference, −$8701.58 [95% CI, −$13 323.56 to −$4079.60]). LTCH closure was not significantly associated with changes in other upstream hospital care patterns (do-not-resuscitate rates, palliative care delivery, tracheostomy rates, or other discharge disposition), or other patient outcomes (hospital LOS, alive and institution-free days, or 90-day mortality) (Figure 2).

**Figure 3.**
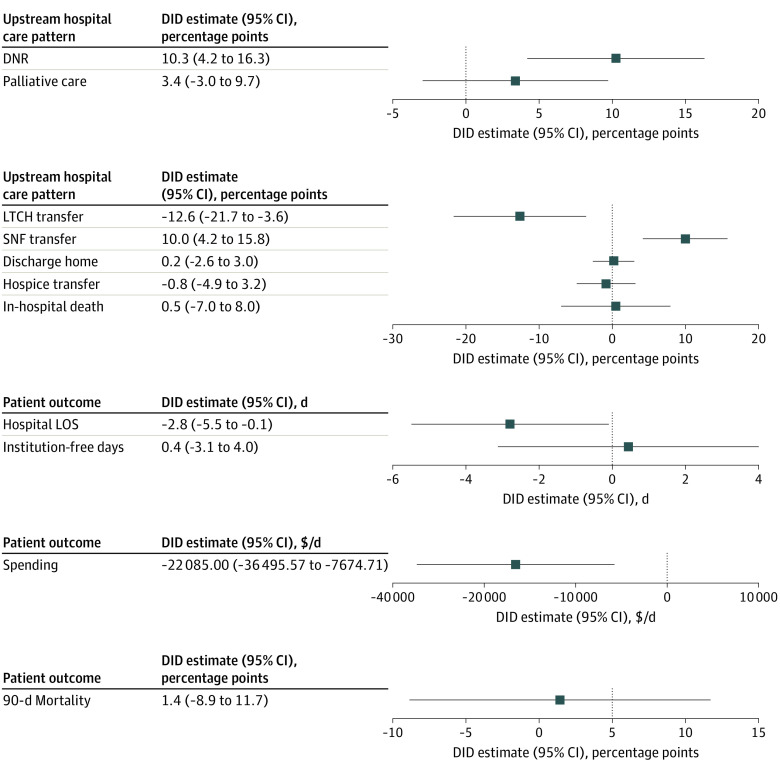
Change in Hospital Care Patterns and Patient Outcomes Associated With LTCH Closure at Closure-Affected Hospitals Compared With Matched Control Hospitals in the Subgroup of Patients Receiving a Tracheostomy Estimates and 95% CIs of change in outcomes were generated by difference-in-differences (DID) analysis adjusted for hospital- and patient-level characteristics, with matched exposure-control hospital pairs as random effect. DNR indicates do-not-resuscitate; LOS, length of stay; LTCH, long-term acute care hospital; SNF, skilled nursing facility; IFD, alive and institution-free days.

Among the subgroup of patients receiving a tracheostomy, LTCH closure was similarly associated with decreased LTCH transfer (difference, −12.6 [95% CI, −21.7 to −3.6] percentage points) and decreased total spending per days alive within 90 days (difference, −$22 085.00 [95% CI, −$36 495.57 to −$7674.71]). Additionally, LTCH closure was also associated with increased do-not-resuscitate rates (difference, 10.3 [95% CI, 4.2 to 16.3] percentage points) and increased SNF transfer (difference, 10.0 [95% CI, 4.2 to 15.8] percentage points), but not other changes in upstream care patterns or mortality measures (Figure 3).

In falsification testing, there was no association between LTCH closure and the occurrence of septic shock or the number of chronic comorbidities among patients receiving MV for at least 96 hours and patients receiving a tracheostomy (eTable 5 in Supplement 1). In sensitivity analysis with hospital of admission as a fixed effect, results were similar (eFigure 1 and eFigure 2 in Supplement 1). In sensitivity analyses exploring hospitals with less prior reliance on closing LTCHs (103 hospitals discharging ≥30% of patients receiving a tracheostomy to a closing LTCH and 215 hospitals discharging >0% of patients receiving a tracheostomy to a closing LTCH), point estimates were generally reduced in magnitude (eg, LTCH referral) and lost statistical significance (eg, spending per day alive) (eTables 6-13, eFigure 3, and eFigure 4 in Supplement 1). In staggered difference-in-differences sensitivity analyses, there was no significant difference between closure-affected and matched control hospitals in any of the outcomes in any year prior to exposure (supporting the parallel trends assumption of our primary analyses), and average treatment effects 1 year after LTCH closure were similar to primary analysis estimates (eFigure 5 and eFigure 6 in Supplement 1).

## Discussion

This cohort study used difference-in-differences analysis comparing LTCH closure–affected hospitals with non–closure-affected hospitals to estimate how CMS payment reform and subsequent LTCH closures might affect hospital practice patterns and patient outcomes. While most hospitals remained unaffected by LTCH closure, Closure-affected hospitals had decreased LTCH transfers and spending and increased do-not-resuscitate rates among patients receiving tracheostomy, but there were no significant changes in mortality.

Our study adds to prior literature investigating the association of LTCH use with patient outcomes. While prior studies have assessed the association between LTCHs and outcomes of other patient populations (and similarly found associations between LTCH use and increased spending)^6,27^ using standard multivariable regression adjusting for hospital- and patient-level confounders^27,28^ or instrumental variable analysis (with distance to nearest LTCH,^27^ number of LTCHs in the admitting hospitals’ hospital referral region,^25^ or LTCH openings during prior market expansion as the instrument^6^), we leveraged the closure of LTCHs in difference-in-difference analysis. By defining exposure hospitals solely by their prior reliance on a closing LTCH for postacute care of patients receiving a tracheostomy, we were able to observe how near-total loss of LTCH availability was associated with changing practices and outcomes (among patients with unchanged eligibility for higher CMS reimbursement rates) compared with otherwise similar but unaffected hospitals, while circumventing issues related to the strength of instruments used in prior instrumental variable analyses. Of note, we observed that while almost 20% of LTCHs closed, only approximately 3% of hospitals had relied on a closing LTCH for the postacute care of patients receiving a tracheostomy. The degree to which a hospital relied on a closing LTCH prior to LTCH closure and the degree to which its care patterns were subsequently altered may be related in part to local LTCH prevalence^9,29^: areas with multiple LTCHs^30^ may be more insulated from the effect of a closing LTCH compared with those with zero or 1 LTCH.^31^ Our sensitivity analysis exploring hospitals with less prior reliance on closing LTCHs may suggest a dose-response association, whereby hospitals with higher prior reliance on a single closing LTCH experienced larger changes in LTCH referrals, and the sensitivity of certain outcomes upstream or downstream of LTCH referral (such as spending) may be dependent on the degree of change in LTCH referral.

CMS’s LTCH reimbursement reform, which reserved higher reimbursement for patients admitted to an ICU for 3 days or longer or receiving MV for 96 hours or longer on arrival to the LTCH, was intended to divert other, potentially less complex patients (who previously made up >40% of all LTCH patients^31^) to lower-cost settings, such as SNFs.^6^ However, because reimbursement reform not only led LTCHs to adjust admission criteria but also led many LTCHs to close entirely, we found that closure-affected hospitals changed postacute care discharge practices in unintended ways. Although our cohort would meet CMS criteria for higher LTCH reimbursement rates, we found that patients receiving a tracheostomy were discharged from closure-affected hospitals to SNFs with increased frequency. In the US, tracheostomies are the strongest factor associated with LTCH use over SNF use, as LTCHs are perceived to offer higher levels of interdisciplinary management for complex medical care.^10^ Like LTCHs, SNFs are known to vary in quality and capability of caring for complex patients. While studies comparing SNF and LTCHs in a heterogeneous cohort of inpatients who were not critically ill admitted to a general medical service found similar clinical outcomes between SNFs and LTCHs,^10,27^ the ability of SNFs in the US to care for patients receiving or recovering from prolonged MV in the US is understudied.^32,33^ Our findings suggest that discharge of a subset of patients recovering from prolonged MV (eg, after weaning or while receiving stable MV) to SNFs may result in similar mortality outcomes with lower spending compared with LTCHs, although functional outcomes, complication rates, and other patient-centered outcomes remain unclear and require further study. Of note, we observed a surprising decrease in LOS among patients receiving tracheostomy in the context of changing discharge locations, which may reflect relatively longer wait times for LTCH bed availability after patient readiness for discharge (especially in areas with low LTCH prevalence) compared with SNF beds.

Among patients receiving a tracheostomy, we observed an increase in do-not-resuscitate rates associated with LTCH closure. A 2012 study by Stelfox et al^34^ found that reduced ICU bed availability was associated with an increased likelihood of change in patient goals of care, without significant change in hospital mortality. The plasticity of end-of-life decisions to changes in available postacute resources has not been clearly demonstrated. Whether increased do-not-resuscitate orders after LTCH closure represent improved attainment of goal-concordant care or are the result of institutional pressure resulting from changing discharge options remains unclear. Furthermore, while we observed an increase in do-not-resuscitate orders among patients receiving tracheostomies, we did not observe this increase among the larger cohort of patients receiving MV for 96 hours or longer, nor a change in rates of tracheostomy placement. It is possible that transitions in goals of care occur gradually over a longer period among patients who are more severely ill, and tracheostomy decisions may occur before patients and families are ready to transition goals or the tracheostomy may be viewed by patients and families as part of a time-limited trial that informs later goals-of-care discussions.

Our estimates of spending are based on all payments due (including out-of-pocket payments by the beneficiary and other payer payments) for care received at hospitals, SNFs, and LTCHs. As in prior work,^7^ lower total spending is likely driven by the lower prospective payment rate to hospitals and SNFs compared with LTCHs. However, the costs incurred by hospitals and SNFs (over the reimbursed prospective payment fee) may increase as LTCH use decreases; indeed, in the early 2000s, use of LTCHs for postacute care was suggested as a cost-saving measure from the perspective of hospitals.^1^ Future studies should investigate how costs to hospitals and SNFs and the overall economic impact on society have changed with LTCH closures.

### Limitations

Our study has limitations. First, analyses are based on administrative data and may be vulnerable to misclassification. However, we used previously validated and published *ICD-9* and *ICD-10* algorithms^24,35^ that have been verified for continuity using CMS General Equivalence Mappings. *ICD-9* and *ICD-10* codes are known to slightly overestimate the true incidence of patients receiving MV for 96 hours or longer^36^ and underestimate do-not-resuscitate orders and palliative care delivery,^37,38^ but performance characteristics are unlikely to be differential across closure-affected and control hospitals. Our claims-based algorithm to adjust for acute severity of illness has also been validated against the Sequential Organ Failure Assessment score with strong performance characteristics.^24^ Second, we examined a number of outcomes that are known to be patient-centered^39^ and/or important to understanding health system resource use^2^; however, other outcomes, such as attainment of goal-concordant care, patient and family satisfaction, and functional outcomes (including tracheostomy decannulation, mobility, cognitive, and psychiatric outcomes), will be important to assess via other study designs and databases. Third, our analysis was limited to Medicare beneficiaries; analyses of younger cohorts may yield different estimates. Fourth, LTCHs have a wide spectrum of relationships with short-stay hospitals; some may be colocated or co-owned, while others are completely independent. We grouped all sites defined by CMS as LTCHs together in our analysis (ie, LTCHs that are eligible for a higher prospective payment and are subject to CMS payment reform); it is possible that subgroup analysis by LTCH ownership or colocation may lead different estimates. Fifth, a loss of an LTCH could have been accompanied by a gain of another LTCH, thereby biasing our results toward the null. Sixth, the latest year hospital practices and outcomes were assessed was 2019 (for LTCHs closing in 2018), although the full impact of payment reform led to LTCH closures through 2020. Future studies including hospital practices through 2021 (which may additionally be influenced by COVID-19) may be needed to update our findings.

## Conclusions

This cohort study found that after CMS reimbursement reform, LTCH closure was associated with changes in upstream hospital care practices, including decreased LTCH transfer, increased do-not-resuscitate rates, and decreased overall spending but was not associated with changes in mortality, LOS, or alive and institution-free days. Further studies are needed to understand how LTCH closure may be associated with other important outcomes, including patient and family satisfaction, functional outcomes, and attainment of goal-concordant care.

## Notes

Supplement 1. **eTable 1.** Characteristics of Closure-Affected and Control hospitals **eTable 2.** Characteristics of Patients Receiving MV ≥96 at Closure-Affected and Control Hospitals, Before and After Hospital-Level Matching **eTable 3.** Baseline Characteristics of Patients Receiving a Tracheostomy at Closure-Affected Hospitals and Matched Control Hospitals, After Hospital-Level Matching and Selection of Preclosure and Postclosure Years **eTable 4.** Unadjusted Outcomes of Patients Receiving a Tracheostomy at Closure-Affected and Matched Control Hospitals **eTable 5.** Falsification Testing **eTable 6.** Hospital Characteristics of Sensitivity Analysis, Defining Closure-Affected Hospitals as Those Discharging >30% of Patients Receiving a Tracheostomy to a Closing LTCH **eTable 7.** Hospital Characteristics of Sensitivity Analysis, Defining Closure-Affected Hospitals as Those Discharging >0% of Patients Receiving a Tracheostomy to a Closing LTCH **eTable 8.** Baseline Patient Characteristics of Patients Receiving MV ≥96 Hours in Sensitivity Analysis, Defining Closure-Affected Hospitals as Those Discharging ≥30% of Patients Receiving a Tracheostomy to a Closing LTCH **eTable 9.** Baseline Patient Characteristics of Patients Receiving a Tracheostomy in Sensitivity Analysis, Defining Closure-Affected Hospitals as Those Discharging ≥30% of Patients Receiving a Tracheostomy to a Closing LTCH **eTable 10.** Baseline Patient Characteristics of Patients Receiving MV ≥96 Hours in Sensitivity Analysis, Defining Closure-Affected Hospitals as Those Discharging ≥0% of Patients Receiving a Tracheostomy to a Closing LTCH **eTable 11.** Baseline Patient Characteristics of Patients Receiving a Tracheostomy in Sensitivity Analysis, Defining Closure-Affected Hospitals as Those Discharging ≥0% of Patients Receiving a Tracheostomy to a Closing LTCH **eTable 12.** Unadjusted Outcomes in Sensitivity Analysis, Defining Closure-Affected Hospitals as Those Discharging ≥30% of Patients Receiving a Tracheostomy to a Closing LTCH **eTable 13.** Unadjusted Outcomes in Sensitivity Analysis, Defining Closure-Affected Hospitals as Those Discharging ≥0% of Patients Receiving a Tracheostomy to a Closing LTCH **eFigure 1.** Sensitivity Analysis With Hospital of Admission as Fixed Effect, Among Patients Receiving Mechanical Ventilation ≥96 Hours **eFigure 2.** Sensitivity Analysis With Hospital of Admission as Fixed Effect, Among Patients Receiving a Tracheostomy **eFigure 3.** Adjusted Outcomes in Sensitivity Analysis Varying Definition of Closure-Affected Hospital, Among Patients Receiving MV ≥96 Hours **eFigure 4.** Adjusted Outcomes in Sensitivity Analysis Varying Definition of Closure-Affected Hospital, Among Patients Receiving a Tracheostomy **eFigure 5.** Adjusted Outcomes in Sensitivity Analysis Using the Callaway/Sant’Anna Staggered Difference-in- Difference Method Among Patients Receiving MV ≥96 Hours **eFigure 6.** Adjusted Outcomes in Sensitivity Analysis Using the Callaway/Sant’Anna Staggered Difference-in-Difference Method Among Patients Receiving a Tracheostomy

Supplement 2.
Data Sharing Statement

## References

[1] Seneff MG, Wagner D, Thompson D, Honeycutt C, Silver MR. The impact of long-term acute-care facilities on the outcome and cost of care for patients undergoing prolonged mechanical ventilation. Crit Care Med. 2000;28(2):342-350. doi:10.1097/00003246-200002000-00009 10708164

[2] Cox CE, Carson SS. Medical and economic implications of prolonged mechanical ventilation and expedited post-acute care. Semin Respir Crit Care Med. 2012;33(4):357-361. doi:10.1055/s-0032-1321985 22875381

[3] Law AC, Stevens JP, Choi E, . Days out of institution after tracheostomy and gastrostomy placement in critically ill older adults. Ann Am Thorac Soc. 2022;19(3):424-432. doi:10.1513/AnnalsATS.202106-649OC 34388080 PMC8937225

[4] Rak KJ, Ashcraft LE, Kuza CC, . Effective care practices in patients receiving prolonged mechanical ventilation: an ethnographic study. Am J Respir Crit Care Med. 2020;201(7):823-831. doi:10.1164/rccm.201910-2006OC 32023081 PMC7124716

[5] Kahn JM, Davis BS, Le TQ, Yabes JG, Chang CH, Angus DC. Variation in mortality rates after admission to long-term acute care hospitals for ventilator weaning. J Crit Care. 2018;46:6-12. doi:10.1016/j.jcrc.2018.03.022 29627660 PMC6014911

[6] Einav L, Finkelstein A, Mahoney N. *Long-Term Care Hospitals: A Case Study in Waste*. National Bureau of Economic Research; 2021.

[7] Kahn JM, Benson NM, Appleby D, Carson SS, Iwashyna TJ. Long-term acute care hospital utilization after critical illness. JAMA. 2010;303(22):2253-2259. doi:10.1001/jama.2010.761 20530778 PMC3094575

[8] Medicare Payment Advisory Commission. March 2018 Report to the Congress: Medicare Payment Policy. Accessed October 25, 2023. https://www.medpac.gov/document/http-www-medpac-gov-docs-default-source-reports-mar18_medpac_entirereport_sec_rev_0518-pdf/

[9] Kahn JM, Werner RM, Carson SS, Iwashyna TJ. Variation in long-term acute care hospital use after intensive care. Med Care Res Rev. 2012;69(3):339-350. doi:10.1177/1077558711432889 22311957 PMC5503694

[10] Makam AN, Nguyen OK, Xuan L, Miller ME, Goodwin JS, Halm EA. Factors associated with variation in long-term acute care hospital vs skilled nursing facility use among hospitalized older adults. JAMA Intern Med. 2018;178(3):399-405. doi:10.1001/jamainternmed.2017.8467 29404575 PMC5840036

[11] Centers for Medicare and Medicaid Services. Chronic conditions data warehouse. Accessed October 26, 2020. https://www2.ccwdata.org/web/guest/condition-categories

[12] Kahn JM, Carson SS, Angus DC, Linde-Zwirble WT, Iwashyna TJ. Development and validation of an algorithm for identifying prolonged mechanical ventilation in administrative data. Health Serv Outcomes Res Methodol. 2009;9(2):117-132. doi:10.1007/s10742-009-0050-6

[13] Cox CE, Carson SS, Holmes GM, Howard A, Carey TS. Increase in tracheostomy for prolonged mechanical ventilation in North Carolina, 1993-2002. Crit Care Med. 2004;32(11):2219-2226. doi:10.1097/01.CCM.0000145232.46143.40 15640633

[14] Cox CE, Martinu T, Sathy SJ, . Expectations and outcomes of prolonged mechanical ventilation. Crit Care Med. 2009;37(11):2888-2894. doi:10.1097/CCM.0b013e3181ab86ed 19770733 PMC2766420

[15] Law AC, Tian W, Song Y, Stevens JP, Walkey AJ. Decline in prolonged acute mechanical ventilation, 2011-2019. Am J Respir Crit Care Med. 2022;206(5):640-644. doi:10.1164/rccm.202203-0473LE 35608537 PMC9716908

[16] Zilberberg MD, Luippold RS, Sulsky S, Shorr AF. Prolonged acute mechanical ventilation, hospital resource utilization, and mortality in the United States. Crit Care Med. 2008;36(3):724-730. doi:10.1097/CCM.0B013E31816536F7 18209667

[17] Kahn JM, Iwashyna TJ. Accuracy of the discharge destination field in administrative data for identifying transfer to a long-term acute care hospital. BMC Res Notes. 2010;3(1):205. doi:10.1186/1756-0500-3-205 20663175 PMC2917437

[18] Johnston SC. Combining ecological and individual variables to reduce confounding by indication: case study—subarachnoid hemorrhage treatment. J Clin Epidemiol. 2000;53(12):1236-1241. doi:10.1016/S0895-4356(00)00251-1 11146270

[19] Frank B. Definitions of “cost” in Medicare utilization files. Accessed October 7, 2022. https://resdac.org/sites/datadocumentation.resdac.org/files/Definitions%20of%20%27Cost%27%20in%20Medicare%20Utilization%20Files%20%28Slides%29.pdf

[20] Mendez CM, Harrington DW, Christenson P, Spellberg B. Impact of hospital variables on case mix index as a marker of disease severity. Popul Health Manag. 2014;17(1):28-34. doi:10.1089/pop.2013.0002 23965045 PMC3931432

[21] Warton EM, Parker M, Karter AJ. How D-I-D you do that: basic difference-in-differences models in SAS. Paper presented at: Western Users of SAS Software Conference; September 7, 2016. San Francisco, CA.

[22] Centers for Disease Control and Prevention; Agency for Toxic Substances and Disease Registry; Geospatial Research, Analysis, and Services Program. CDC/ATSDR Social Vulnerability Index 2010 database, United States. Accessed February 28, 2022. https://www.atsdr.cdc.gov/placeandhealth/svi/data_documentation_download.html

[23] Healthcare Cost and Utilization Project. Procedure classes for *ICD-9-CM*. Accessed January 15, 2017. https://www.hcup-us.ahrq.gov/toolssoftware/procedure/procedure.jsp

[24] Bosch NA, Law AC, Rucci J, Peterson D, Walkey AJ. Predictive validity of the sequential organ failure assessment score versus claims-based scores among critically ill patients. Ann Am Thorac Soc. 2022;19(6):1072-1076. doi:10.1513/AnnalsATS.202111-1251RL 35266866 PMC9797032

[25] Kahn JM, Werner RM, David G, Ten Have TR, Benson NM, Asch DA. Effectiveness of long-term acute care hospitalization in elderly patients with chronic critical illness. Med Care. 2013;51(1):4-10. doi:10.1097/MLR.0b013e31826528a7 22874500 PMC3500575

[26] Callaway B, Sant’Anna PHC. Difference-in-Differences with multiple time periods. J Econom. 2021;225(2):200-230. doi:10.1016/j.jeconom.2020.12.001

[27] Makam AN, Nguyen OK, Miller ME, Shah SJ, Kapinos KA, Halm EA. Comparative effectiveness of long-term acute care hospital versus skilled nursing facility transfer. BMC Health Serv Res. 2020;20(1):1032. doi:10.1186/s12913-020-05847-6 33176767 PMC7656509

[28] Mehta AB, Matlock D, Douglas IS. Association of proximity to a long-term acute care hospital with hospital tracheostomy practices. Crit Care Med. 2022;50(1):93-102. doi:10.1097/CCM.0000000000005146 34166292 PMC9078375

[29] Makam AN, Nguyen OK, Xuan L, Miller ME, Halm EA. Long-term acute care hospital use of non-mechanically ventilated hospitalized older adults. J Am Geriatr Soc. 2018;66(11):2112-2119. doi:10.1111/jgs.15564 30295927 PMC6239216

[30] Medicare Payment Advisory Commission. March 2014 Report to the Congress: Medicare Payment Policy. Accessed October 25, 2023. https://www.medpac.gov/document/report-to-the-congress-medicare-payment-policy-march-2014/

[31] Makam AN, Nguyen OK, Kirby B, Miller ME, Xuan L, Halm EA. Effect of site-neutral payment policy on long-term acute care hospital use. J Am Geriatr Soc. 2018;66(11):2104-2111. doi:10.1111/jgs.15539 30281783 PMC6382068

[32] Goodwin JS, Li S, Middleton A, Ottenbacher K, Kuo YF. Differences between skilled nursing facilities in risk of subsequent long-term care placement. J Am Geriatr Soc. 2018;66(10):1880-1886. doi:10.1111/jgs.15377 29656399 PMC6181774

[33] Keohane LM, Mart MF, Ely EW, . Establishing Medicaid incentives for liberating nursing home patients from ventilators. J Am Geriatr Soc. 2022;70(1):259-268. doi:10.1111/jgs.17513 34668195 PMC8742752

[34] Stelfox HT, Hemmelgarn BR, Bagshaw SM, . Intensive care unit bed availability and outcomes for hospitalized patients with sudden clinical deterioration. Arch Intern Med. 2012;172(6):467-474. doi:10.1001/archinternmed.2011.2315 22412076

[35] Vail EA, Wunsch H, Pinto R, . Use of Hydrocortisone, ascorbic acid, and thiamine in adults with septic shock. Am J Respir Crit Care Med. 2020;202(11):1531-1539. doi:10.1164/rccm.202005-1829OC 32706593

[36] Wunsch H, Kramer A, Gershengorn HB. Validation of intensive care and mechanical ventilation codes in Medicare data. Crit Care Med. 2017;45(7):e711-e714. doi:10.1097/CCM.0000000000002316 28403118 PMC6557134

[37] Fonseca L, Walkey AJ, Ma X, Hua M. Validation of the V49.86 code for do-not-resuscitate status in hospitalized patients at a single academic medical center. Ann Am Thorac Soc. 2018;15(10):1234-1237. doi:10.1513/AnnalsATS.201804-257RL 29944385 PMC6321991

[38] Hua M, Li G, Clancy C, Morrison RS, Wunsch H. Validation of the V66.7 code for palliative care consultation in a single academic medical center. J Palliat Med. 2017;20(4):372-377. doi:10.1089/jpm.2016.0363 27925839 PMC5385421

[39] Groff AC, Colla CH, Lee TH. days spent at home—a patient-centered goal and outcome. N Engl J Med. 2016;375(17):1610-1612. doi:10.1056/NEJMp1607206 27783911 PMC5996758

