# Supplementary material for: Patient Outcomes After Long-Term Acute Care Hospital Closures
Source: JAMA Netw Open. 2023 Nov 21;6(11):e2344377. doi: 10.1001/jamanetworkopen.2023.44377 (PMC10663966; doi:10.1001/jamanetworkopen.2023.44377)
Supplement: Supplement 1. — eTable 1. Characteristics of Closure-Affected and Control hospitals eTable 2. Characteristics of Patients Receiving MV ≥96 at Closure-Affected and Control Hospitals, Before and After Hospital-Level Matching eTable 3. Baseline Characteristics of Patients Receiving a Tracheostomy at Closure-Affected Hospitals and Matched Control Hospitals, After Hospital-Level Matching and Selection of Preclosure and Postclosure Years eTable 4. Unadjusted Outcomes of Patients Receiving a Tracheostomy at Closure-Affected and Matched Control Hospitals eTable 5. Falsification Testing eTable 6. Hospital Characteristics of Sensitivity Analysis, Defining Closure-Affected Hospitals as Those Discharging >30% of Patients Receiving a Tracheostomy to a Closing LTCH eTable 7. Hospital Characteristics of Sensitivity Analysis, Defining Closure-Affected Hospitals as Those Discharging >0% of Patients Receiving a Tracheostomy to a Closing LTCH eTable 8. Baseline Patient Characteristics of Patients Receiving MV ≥96 Hours in Sensitivity Analysis, Defining Closure-Affected Hospitals as Those Discharging ≥30% of Patients Receiving a Tracheostomy to a Closing LTCH eTable 9. Baseline Patient Characteristics of Patients Receiving a Tracheostomy in Sensitivity Analysis, Defining Closure-Affected Hospitals as Those Discharging ≥30% of Patients Receiving a Tracheostomy to a Closing LTCH eTable 10. Baseline Patient Characteristics of Patients Receiving MV ≥96 Hours in Sensitivity Analysis, Defining Closure-Affected Hospitals as Those Discharging ≥0% of Patients Receiving a Tracheostomy to a Closing LTCH eTable 11. Baseline Patient Characteristics of Patients Receiving a Tracheostomy in Sensitivity Analysis, Defining Closure-Affected Hospitals as Those Discharging ≥0% of Patients Receiving a Tracheostomy to a Closing LTCH eTable 12. Unadjusted Outcomes in Sensitivity Analysis, Defining Closure-Affected Hospitals as Those Discharging ≥30% of Patients Receiving a Tracheostomy to a Closing LTCH eTable 13. Unadjusted [file jamanetwopen-e2344377-s001.pdf]

Supplemental Online Content

Law AC, Bosch NA, Song Y, et al. Patient outcomes after long-term acute care hospital closures. *JAMA Netw Open*. 2023;6(11):e2344377. doi:10.1001/jamanetworkopen.2023.44377

eTable 1. Characteristics of Closure-Affected and Control hospitals
eTable 2. Characteristics of Patients Receiving MV ≥96 at Closure-Affected and Control Hospitals, Before and After Hospital-Level Matching
eTable 3. Baseline Characteristics of Patients Receiving a Tracheostomy at Closure-Affected Hospitals and Matched Control Hospitals, After Hospital-Level Matching and Selection of Preclosure and Postclosure Years
eTable 4. Unadjusted Outcomes of Patients Receiving a Tracheostomy at Closure-Affected and Matched Control Hospitals
eTable 5. Falsification Testing
eTable 6. Hospital Characteristics of Sensitivity Analysis, Defining Closure-Affected Hospitals as Those Discharging >30% of Patients Receiving a Tracheostomy to a Closing LTCH
eTable 7. Hospital Characteristics of Sensitivity Analysis, Defining Closure-Affected Hospitals as Those Discharging >0% of Patients Receiving a Tracheostomy to a Closing LTCH
eTable 8. Baseline Patient Characteristics of Patients Receiving MV ≥96 Hours in Sensitivity Analysis, Defining Closure-Affected Hospitals as Those Discharging ≥30% of Patients Receiving a Tracheostomy to a Closing LTCH
eTable 9. Baseline Patient Characteristics of Patients Receiving a Tracheostomy in Sensitivity Analysis, Defining Closure-Affected Hospitals as Those Discharging ≥30% of Patients Receiving a Tracheostomy to a Closing LTCH
eTable 10. Baseline Patient Characteristics of Patients Receiving MV ≥96 Hours in Sensitivity Analysis, Defining Closure-Affected Hospitals as Those Discharging ≥0% of Patients Receiving a Tracheostomy to a Closing LTCH
eTable 11. Baseline Patient Characteristics of Patients Receiving a Tracheostomy in Sensitivity Analysis, Defining Closure-Affected Hospitals as Those Discharging ≥0% of Patients Receiving a Tracheostomy to a Closing LTCH
eTable 12. Unadjusted Outcomes in Sensitivity Analysis, Defining Closure-Affected Hospitals as Those Discharging ≥30% of Patients Receiving a Tracheostomy to a Closing LTCH
eTable 13. Unadjusted Outcomes in Sensitivity Analysis, Defining Closure-Affected Hospitals as Those Discharging ≥0% of Patients Receiving a Tracheostomy to a Closing LTCH
eFigure 1. Sensitivity Analysis With Hospital of Admission as Fixed Effect, Among Patients Receiving Mechanical Ventilation ≥96 Hours
eFigure 2. Sensitivity Analysis With Hospital of Admission as Fixed Effect, Among Patients Receiving a Tracheostomy
eFigure 3. Adjusted Outcomes in Sensitivity Analysis Varying Definition of Closure-Affected Hospital, Among Patients Receiving MV ≥96 Hours
eFigure 4. Adjusted Outcomes in Sensitivity Analysis Varying Definition of Closure-Affected Hospital, Among Patients Receiving a Tracheostomy
eFigure 5. Adjusted Outcomes in Sensitivity Analysis Using the Callaway/Sant'Anna Staggered Difference-in-Difference Method Among Patients Receiving MV ≥96 Hours
eFigure 6. Adjusted Outcomes in Sensitivity Analysis Using the Callaway/Sant'Anna Staggered Difference-in-Difference Method Among Patients Receiving a Tracheostomy

This supplemental material has been provided by the authors to give readers additional information about their work.

© 2023 Law AC et al. *JAMA Network Open.*

Table S1. Characteristics of Closure-affected and Control hospitals. Characteristics of the full pool of Closure-affected hospitals and Control Hospitals are shown with standardized mean differences on the left half of the table; characteristics after selection of matched control hospitals (by matching on hospital characteristics and then selecting years before and after LTCH closure) are shown on the right half of the table. *Prior LTCH use = % of patients receiving tracheostomy discharged to any LTCH in the year prior to LTCH closure.

**Hospital Characteristics**	**All Closure-affected Hospitals N = 46 hospitals**	**All Control Hospitals N = 1031 hospitals**	**SMD**	**Matched Closure-affected Hospitals N = 45 hospitals**	**Matched Control Hospitals N = 45 hospitals**	**SMD**
Hospital Bed Number						
Mean±SD	371.6±175.3	363.6±246.9	0.04	365.2±171.8	362.4±194.2	.02
Median (Q1, Q3)	345.0 (244.0,445.0)	302.0 (217.0,427.0)		343.0 (244.0,444.0)	315.0 (202.0,482.0)	
Ownership, N (%)						
For Profit	6 (13.0%)	206 (20.2%)	-0.19	6 (13.3%)	6 (13.3%)	0.0
Private Nonprofit	35 (76.1%)	700 (68.7%)	0.17	35 (77.8%)	35 (77.8%)	0.0
Public	5 (10.9%)	113 (11.1%)	-0.01	4 (8.9%)	4 (8.9%)	0.0
Teaching hospital, N (%)	38 (82.6%)	692 (67.9%)	0.35	37 (82.2%)	37 (82.2%)	0.0
Safety net hospital, N (%)	10 (22.2%)	283 (27.8%)	-0.13	10 (22.2%)	10 (22.2%)	0.0
Hospital Region, N (%)						
Northeast	3 (6.5%)	171 (16.8%)	-0.32	3 (6.7%)	3 (6.7%)	0.0
Midwest	6 (13.0%)	131 (12.9%)	0.01	6 (13.3%)	6 (13.3%)	0.0
South	34 (73.9%)	499 (49.0%)	0.53	33 (73.3%)	33 (73.3%)	0.0
West	3 (6.5%)	218 (21.4%)	-0.44	3 (6.7%)	3 (6.7%)	0.0
Prior LTCH use*						
Mean±SD	0.61±0.28	0.61±0.27	-0.03	0.62±0.28	0.68±0.25	-.23
Median (Q1, Q3)	0.60 (0.33,0.82)	0.64 (0.41,0.83)		0.61 (0.33,0.82)	0.71 (0.57,0.83)	
Exposure Year, N (%)						
2012	-	-		6 (13.3%)	6 (13.3%)	0.0
2013	-	-		7 (15.6%)	7 (15.6%)	0.0
2014	-	-		4 (8.9%)	4 (8.9%)	0.0
2015	-	-		1 (2.2%)	1 (2.2%)	0.0
2016	-	-		2 (4.4%)	2 (4.4%)	0.0
2017	-	-		8 (17.8%)	8 (17.8%)	0.0
2018	-	-		17 (37.8%)	17 (37.8%)	0.0

© 2023 Law AC et al. *JAMA Network Open.*

Table S2. Characteristics of patients receiving MV:=96 at Closure-affected and Control hospitals, before and after hospital-level matching. Characteristics of patients from the full pool of Closure-affected hospitals and Control Hospitals from 2011-2019 are shown with standardized mean differences on the left half of the table; characteristics of patients from selected matched hospitals, 2011-2019, are shown on the right half of the table. Characteristics of matched hospital after restriction to pre- and post-years are shown in Table 1 of the main manuscript.

**Patient Characteristics N (%) unless otherwise indicated**	**Patients at ALL Closure-affected Hospitals (N = 19,679 patients at 46 hospitals)**	**Patients at ALL Control Hospitals (N = 437,923 patients at 1031 hospitals)**	**SMD**	**Patients at MATCHED Closure-affected Hospitals (N = 19,294 patients at 45 hospitals)**	**Patients at MATCHED Control Hospitals (N = 19,245 patients at 45 hospitals)**	**SMD**
Age, Mean±SD	76.4±7.3	76.8±7.5	-0.05	76.5±7.3 (19294)	76.2±7.2 (19245)	0.03
Female	9521 (48.4%)	208132 (47.5%)	0.02	9372 (48.6%)	8979 (46.7%)	0.04
Race						
White non-Hispanic	14732 (74.9%)	344689 (78.7%)	-0.09	14415 (74.7%)	16120 (83.8%)	-0.23
Black non-Hispanic	4022 (20.4%)	58602 (13.4%)	0.19	3972 (20.6%)	2163 (11.2%)	0.26
Other	925 (4.7%)	34632 (7.9%)	-0.13	907 (4.7%)	962 (5.0%)	-0.01
Social vulnerability index, Mean±SD**	0.5±0.3	0.5±0.3	-0.09	0.5±0.3 (19294)	0.5±0.3 (19245)	-0.01
Dual Eligibility	4114 (20.9%)	107875 (24.6%)	-0.09	4084 (21.2%)	4051 (21.0%)	0.00
Surgical patient	8539 (43.4%)	192241 (43.9%)	-0.01	8415 (43.6%)	8316 (43.2%)	0.01
Comorbidities, selected						
Alzheimer's disease/dementia	6955 (35.3%)	148957 (34.0%)	0.03	6885 (35.7%)	5975 (31.0%)	0.10
Atrial fibrillation	7135 (36.3%)	161863 (37.0%)	-0.02	7004 (36.3%)	7333 (38.1%)	-0.04
Cancer	3774 (19.2%)	79469 (18.1%)	0.03	3609 (18.7%)	3555 (18.5%)	0.01
Congestive heart failure	13624 (69.2%)	309825 (70.7%)	-0.03	13446 (69.7%)	13120 (68.2%)	0.03
Chronic kidney disease	15514 (78.8%)	340485 (77.7%)	0.03	15201 (78.8%)	14796 (76.9%)	0.05
Chronic obstructive pulmonary disease and bronchiectasis	10596 (53.8%)	226888 (51.8%)	0.04	10463 (54.2%)	10040 (52.2%)	0.04
Depressive disorders	7016 (35.7%)	151243 (34.5%)	0.02	6898 (35.8%)	6727 (35.0%)	0.02
Diabetes	11038 (56.1%)	242009 (55.3%)	0.02	10889 (56.4%)	10416 (54.1%)	0.05
Hip/pelvic fracture	662 (3.4%)	14915 (3.4%)	0.00	655 (3.4%)	660 (3.4%)	0.00
Hyperlipidemia	13519 (68.7%)	295042 (67.4%)	0.03	13293 (68.9%)	13148 (68.3%)	0.01
Hypertension	18134 (92.1%)	401203 (91.6%)	0.02	17786 (92.2%)	17631 (91.6%)	0.02
Ischemic heart disease	14074 (71.5%)	314288 (71.8%)	-0.01	13828 (71.7%)	13495 (70.1%)	0.03
Stroke/transient ischemic attack	5258 (26.7%)	117601 (26.9%)	0.00	5204 (27.0%)	4917 (25.5%)	0.03
Acute organ dysfunction						
Cardiovascular	11007 (55.9%)	237150 (54.2%)	0.04	10782 (55.9%)	10317 (53.6%)	0.05
Neurologic	7174 (36.5%)	167103 (38.2%)	-0.04	7050 (36.5%)	7503 (39.0%)	-0.05
Hematologic	4795 (24.4%)	96296 (22.0%)	0.06	4633 (24.0%)	4412 (22.9%)	0.03
Hepatic	1463 (7.4%)	33527 (7.7%)	-0.01	1405 (7.3%)	1478 (7.7%)	-0.02
Renal	11963 (60.8%)	263496 (60.2%)	0.01	11710 (60.7%)	11389 (59.2%)	0.03
Hospital size						
Small (1 - 200)	2021 (10.3%)	47901 (11.1%)	-0.03	2021 (10.5%)	2364 (12.3%)	-0.06
Medium (201 - 400)	7137 (36.3%)	179594 (41.8%)	-0.11	7137 (37.0%)	5761 (29.9%)	0.15
Large (> 400)	10521 (53.5%)	202349 (47.1%)	0.13	10136 (52.5%)	11120 (57.8%)	-0.11

© 2023 Law AC et al. *JAMA Network Open.*

Patient Characteristics N (%) unless otherwise indicated	Patients at ALL Closure-affected Hospitals (N = 19,679 patients at 46 hospitals)	Patients at ALL Control Hospitals (N = 437,923 patients at 1031 hospitals)	SMD	Patients at MATCHED Closure-affected Hospitals (N = 19,294 patients at 45 hospitals)	Patients at MATCHED Control Hospitals (N = 19,245 patients at 45 hospitals)	SMD
Ownership						
For Profit	1856 (9.4%)	69460 (16.2%)	-0.20	1856 (9.6%)	1741 (9.0%)	0.02
Private Nonprofit	16262 (82.6%)	310841 (72.3%)	0.25	16262 (84.3%)	15684 (81.5%)	0.07
Public	1561 (7.9%)	49543 (11.5%)	-0.12	1176 (6.1%)	1820 (9.5%)	-0.13
Teaching hospital	17586 (89.4%)	331763 (77.2%)	0.33	17201 (89.2%)	16677 (86.7%)	0.08
Safety net hospital	5532 (28.7%)	133790 (31.2%)	-0.05	5532 (28.7%)	4695 (24.4%)	0.10
Hospital Region						
Northeast	872 (4.4%)	82822 (19.3%)	-0.47	872 (4.5%)	2099 (10.9%)	-0.24
Midwest	2198 (11.2%)	55532 (12.9%)	-0.05	2198 (11.4%)	3128 (16.3%)	-0.14
South	15717 (79.9%)	212327 (49.4%)	0.67	15332 (79.5%)	13237 (68.8%)	0.25
West	892 (4.5%)	79163 (18.4%)	-0.45	892 (4.6%)	781 (4.1%)	0.03
Prior LTCH use*						
Mean±SD	0.59±0.28	0.57±0.27	0.07	0.59±0.28 (19294)	0.65±0.22 (19245)	-0.24
Median (IQR)	0.67 (0.27,0.82)	0.60 (0.37,0.75)		0.67 (0.33,0.82)	0.71 (0.50,0.82)	
Rural	253 (1.3%)	1445 (0.3%)	0.11	253 (1.3%)	403 (2.1%)	-0.06

© 2023 Law AC et al. *JAMA Network Open.*

Table S3. Baseline characteristics of patients receiving a tracheostomy at Closure-affected hospitals and matched Control hospitals, after hospital-level matching and selection of pre- and post- years. variables were included in multivariable adjustment. *Prior LTCH use = % of patients receiving tracheostomy discharged to any LTCH in the year prior to LTCH closure. **Social Vulnerability Index for each patient's census tract is based on 15 social factors, including poverty, lack of vehicle access, and crowded housing. A census tract's index is a percentile ranking and reflects the proportion of tracts in the country that are equal to or lower in terms of social vulnerability.

	Pre-period			Post-period		
**Characteristics N (%) unless otherwise specified**	**Closure-affected Hospital** (N = 413 patients at 45 hospitals)	**Matched Control Hospital** (N = 405 patients at 45 hospitals)	**SMD**	**Closure-affected Hospital** (N = 341 patients at 45 hospitals)	**Matched Control Hospital** (N = 408 patients at 45 hospitals)	**SMD**
Age, Mean±SD	75.9±6.8	74.9±6.6	0.15	75.4±7.0	74.9±6.1	0.07
Female	206 (49.9%)	200 (49.4%)	0.01	171 (50.1%)	158 (38.7%)	0.23
Race						
White non-Hispanic	302 (73.1%)	331 (81.7%)	-0.21	250 (73.3%)	348 (85.3%)	-0.30
Black non-Hispanic	97 (23.5%)	49 (12.1%)	0.30	77 (22.6%)	41 (10.0%)	0.34
Other	14 (3.4%)	25 (6.2%)	-0.13	14 (4.1%)	19 (4.7%)	-0.03
Social vulnerability index, Mean±SD**	0.5±0.3	0.5±0.3	0.03	0.5±0.3	0.5±0.3	0.01
Dual Eligibility	85 (20.6%)	84 (20.7%)	0.00	63 (18.5%)	66 (16.2%)	0.06
Surgical patient	394 (95.4%)	350 (86.4%)	0.32	302 (88.6%)	355 (87.0%)	0.05
Comorbidities						
Alzheimer's disease/dementia	136 (32.9%)	110 (27.2%)	0.13	112 (32.8%)	121 (29.7%)	0.07
Atrial fibrillation	164 (39.7%)	163 (40.2%)	-0.01	142 (41.6%)	160 (39.2%)	0.05
Cancer	75 (18.2%)	71 (17.5%)	0.02	58 (17.0%)	70 (17.2%)	0.00
Congestive heart failure	315 (76.3%)	276 (68.1%)	0.18	230 (67.4%)	251 (61.5%)	0.12
Chronic kidney disease	330 (79.9%)	308 (76.0%)	0.09	273 (80.1%)	315 (77.2%)	0.07
Chronic obstructive pulmonary disease and bronchiectasis	230 (55.7%)	219 (54.1%)	0.03	190 (55.7%)	207 (50.7%)	0.10
Depressive disorders	143 (34.6%)	152 (37.5%)	-0.06	127 (37.2%)	159 (39.0%)	-0.04
Diabetes	247 (59.8%)	214 (52.8%)	0.14	184 (54.0%)	221 (54.2%)	0.00
Hip/pelvic fracture	12 (2.9%)	12 (3.0%)	0.00	11 (3.2%)	20 (4.9%)	-0.09
Hyperlipidemia	295 (71.4%)	269 (66.4%)	0.11	249 (73.0%)	298 (73.0%)	0.00
Hypertension	388 (93.9%)	375 (92.6%)	0.05	321 (94.1%)	377 (92.4%)	0.07
Ischemic heart disease	308 (74.6%)	264 (65.2%)	0.21	239 (70.1%)	267 (65.4%)	0.10
Stroke/transient ischemic attack	132 (32.0%)	128 (31.6%)	0.01	123 (36.1%)	135 (33.1%)	0.06
Acute organ dysfunction						
Cardiovascular	213 (51.6%)	219 (54.1%)	-0.05	167 (49.0%)	207 (50.7%)	-0.04
Neurologic	140 (33.9%)	146 (36.0%)	-0.05	126 (37.0%)	170 (41.7%)	-0.10
Hematologic	116 (28.1%)	76 (18.8%)	0.22	62 (18.2%)	102 (25.0%)	-0.17
Hepatic	25 (6.1%)	21 (5.2%)	0.04	20 (5.9%)	22 (5.4%)	0.02
Renal	243 (58.8%)	226 (55.8%)	0.06	189 (55.4%)	219 (53.7%)	0.04
Hospital size						
Small (1 - 200)	45 (10.9%)	42 (10.4%)	0.02	26 (7.6%)	37 (9.1%)	-0.05
Medium (201 - 400)	142 (34.4%)	133 (32.8%)	0.03	125 (36.7%)	94 (23.0%)	0.30
Large (> 400)	226 (54.7%)	230 (56.8%)	-0.04	190 (55.7%)	277 (67.9%)	-0.25
Ownership						
For Profit	49 (11.9%)	41 (10.1%)	0.06	34 (10.0%)	20 (4.9%)	0.19
Private Nonprofit	349 (84.5%)	336 (83.0%)	0.04	294 (86.2%)	364 (89.2%)	-0.09

© 2023 Law AC et al. *JAMA Network Open.*

	Pre-period			Post-period		
**Characteristics N (%) unless otherwise specified**	**Closure-affected Hospital** (N = 413 patients at 45 hospitals)	**Matched Control Hospital** (N = 405 patients at 45 hospitals)	**SMD**	**Closure-affected Hospital** (N = 341 patients at 45 hospitals)	**Matched Control Hospital** (N = 408 patients at 45 hospitals)	**SMD**
Public	15 (3.6%)	28 (6.9%)	-0.15	13 (3.8%)	24 (5.9%)	-0.10
Teaching hospital	379 (91.8%)	355 (87.7%)	0.14	315 (92.4%)	358 (87.7%)	0.16
Safety net hospital	182 (44.1%)	90 (22.2%)	0.48	127 (37.2%)	112 (27.5%)	0.21
Hospital Region						
Northeast	40 (9.7%)	48 (11.9%)	-0.07	28 (8.2%)	55 (13.5%)	-0.17
Midwest	54 (13.1%)	62 (15.3%)	-0.06	37 (10.9%)	90 (22.1%)	-0.31
South	308 (74.6%)	274 (67.7%)	0.15	243 (71.3%)	250 (61.3%)	0.21
West	11 (2.7%)	21 (5.2%)	-0.13	33 (9.7%)	13 (3.2%)	0.27
Prior LTCH use*, Median (IQR)	0.60 (0.23,0.80)	0.71 (0.46,0.75)	-	0.56 (0.25,0.78)	0.71 (0.46,0.75)	-
Rural	3 (0.7%)	6 (1.5%)	-0.07	1 (0.3%)	5 (1.2%)	-0.11
Year						
2011	125 (30.3%)	61 (15.1%)	0.37	0 (0.0%)	0 (0.0%)	-
2012	38 (9.2%)	48 (11.9%)	-0.09	0 (0.0%)	0 (0.0%)	-
2013	45 (10.9%)	32 (7.9%)	0.10	104 (30.5%)	70 (17.2%)	0.32
2014	9 (2.2%)	6 (1.5%)	0.05	32 (9.4%)	29 (7.1%)	0.08
2015	12 (2.9%)	21 (5.2%)	-0.12	34 (10.0%)	52 (12.7%)	-0.09
2016	66 (16.0%)	58 (14.3%)	0.05	15 (4.4%)	5 (1.2%)	0.19
2017	118 (28.6%)	179 (44.2%)	-0.33	9 (2.6%)	31 (7.6%)	-0.23
2018	0 (0.0%)	0 (0.0%)	-	47 (13.8%)	42 (10.3%)	0.11
2019	0 (0.0%)	0 (0.0%)	-	100 (29.3%)	179 (43.9%)	-0.31

© 2023 Law AC et al. *JAMA Network Open.*

Table S4. Unadjusted outcomes of patients receiving a Tracheostomy at Closure-affected and matched Control hospitals.

	Pre-period		Post-period	
	**Closure-affected Hospital (N = 413)**	**Matched Control Hospital (N = 405)**	**Closure-affected Hospital (N = 341)**	**Matched Control Hospital (N = 408)**
90-day mortality, N (%)	184 (44.6%)	177 (43.7%)	151 (44.3%)	167 (40.9%)
Palliative care delivery, N (%)	30 (7.3%)	53 (13.1%)	46 (13.5%)	60 (14.7%)
DNR, N (%)	24 (5.8%)	50 (12.3%)	57 (16.7%)	43 (10.5%)
IFD in 90, days				
Mean±SD	7.2±15.7 (413)	8.6±16.1 (405)	8.4±16.4 (341)	10.0±19.3 (408)
Median (IQR)	0.0 (0.0,5.0)	0.0 (0.0,8.0)	0.0 (0.0,8.0)	0.0 (0.0,8.0)
Spending/days alive in 90 days, $				
Mean±SD	183637.30±84836.22 (413)	160167.66±67487.09 (405)	166293.29±63202.86 (341)	181816.02±82136.90 (408)
Median (IQR)	172504.73 (129795.58,221004.00	155773.10 (109493.00,198646.15	163958.40 (116437.82,205061.90	174558.20 (123981.00,223884.76
Index hospital length of stay, days				
Mean±SD	28.6±18.3 (413)	25.3±16.8 (405)	25.9±12.0 (341)	25.7±15.1 (408)
Median (IQR)	24.0 (18.0,33.0)	21.0 (16.0,30.0)	24.0 (18.0,31.0)	22.5 (16.0,32.0)
Hospital disposition, N (%)				
LTCH	229 (55.4%)	252 (62.2%)	147 (43.1%)	276 (67.6%)
SNF or IRF	46 (11.1%)	35 (8.6%)	67 (19.6%)	25 (6.1%)
Hospice (facility or home)	13 (3.1%)	18 (4.4%)	12 (3.5%)	17 (4.2%)
Home	7 (1.7%)	6 (1.5%)	9 (2.6%)	9 (2.2%)
Death	76 (18.4%)	77 (19.0%)	60 (17.6%)	72 (17.6%)

© 2023 Law AC et al. *JAMA Network Open.*

Table S5. Falsification Testing. To assess the validity of difference-in-difference analyses, we conducted a difference-in-difference analysis assessing for an association between LTCH closure and incidence of septic shock (ICD 9: 995.92, 785.52; ICD 10: R6520, R6521) and (b) count of chronic comorbidities. Estimates of association, along with 95% confidence interval and p-value, are shown for primary analysis and sensitivity analyses.

	**Septic Shock**		**Count of chronic comorbidities**	
	**Estimate (95% confidence interval)**	**p-value**	**Estimate (95% confidence interval)**	**p-value**
**Primary analysis:** Hospitals discharging ≥60% of patients receiving a tracheostomy to a closing LTCH in the year prior to LTCH closure	-0.001 (-0.035, 0.033)	0.94	-0.009 (-0.053, 0.035)	0.69
**Sensitivity analysis:** Hospitals discharging ≥30% of patients receiving a tracheostomy to a closing LTCH in the year prior to LTCH closure	0.006 (-0.016, 0.028)	0.60	0.006 (-0.020, 0.032)	0.63
**Sensitivity analysis:** Hospitals discharging >0% of patients receiving a tracheostomy to a closing LTCH in the year prior to LTCH closure	-0.008 (-0.021, 0.005)	0.23	0.004 (-0.014, 0.022)	0.66

© 2023 Law AC et al. *JAMA Network Open.*

Table S6. Hospital characteristics of sensitivity analysis, defining “closure-affected” hospitals as those discharging >30% of patients receiving a tracheostomy to a closing LTCH.

*Prior LTCH use = % of patients receiving tracheostomy discharged to any LTCH in the year prior to LTCH closure.

Hospital Characteristics	Closure-affected Hospitals N = 103 hospitals	Matched Control Hospitals N = 103 hospitals
Hospital Bed Number		
Mean±SD	403.6±233.0	398.9±226.0
Median (Q1, Q3)	357.0 (254.0,508.0)	350.0 (233.0,527.0)
Ownership, N (%)		
For Profit	16 (15.5%)	16 (15.5%)
Private Nonprofit	78 (75.7%)	78 (75.7%)
Public	9 (8.7%)	9 (8.7%)
Teaching hospital, N (%)	82 (79.6%)	82 (79.6%)
Safety net hospital, N (%)	23 (22.3%)	23 (22.3%)
Hospital Region, N (%)		
Northeast	12 (11.7%)	12 (11.7%)
Midwest	14 (13.6%)	14 (13.6%)
South	63 (61.2%)	63 (61.2%)
West	14 (13.6%)	14 (13.6%)
Prior LTCH use*		
Mean±SD	0.65±0.25	0.67±0.24
Median (Q1, Q3)	0.67 (0.50,0.83)	0.71 (0.50,0.86)
Exposure Year, N (%)		
2012	9 (8.7%)	9 (8.7%)
2013	9 (8.7%)	9 (8.7%)
2014	7 (6.8%)	7 (6.8%)
2015	6 (5.8%)	6 (5.8%)
2016	11 (10.7%)	11 (10.7%)
2017	25 (24.3%)	25 (24.3%)
2018	36 (35.0%)	36 (35.0%)

© 2023 Law AC et al. *JAMA Network Open.*

Table S7. Hospital characteristics of sensitivity analysis, defining "closure-affected" hospitals as those discharging >0% of patients receiving a tracheostomy to a closing LTCH.

*Prior LTCH use = % of patients receiving tracheostomy discharged to any LTCH in the year prior to LTCH closure.

Hospital Characteristics	Closure-affected Hospitals N = 215 hospitals	Matched Control Hospitals N = 215 hospitals
Hospital Bed Number		
Mean±SD	466.3±264.0	438.7±248.7
Median (Q1, Q3)	409.0 (280.0,579.0)	365.0 (252.0,609.0)
Ownership, N (%)		
For Profit	33 (15.3%)	33 (15.3%)
Private Nonprofit	159 (74.0%)	159 (74.0%)
Public	23 (10.7%)	23 (10.7%)
Teaching hospital, N (%)	179 (83.3%)	179 (83.3%)
Safety net hospital, N (%)	72 (33.5%)	72 (33.5%)
Hospital Region, N (%)		
Northeast	30 (14.0%)	30 (14.0%)
Midwest	38 (17.7%)	38 (17.7%)
South	125 (58.1%)	125 (58.1%)
West	22 (10.2%)	22 (10.2%)
Prior LTCH use*		
Mean±SD	0.68±0.22	0.69±0.24
Median (Q1, Q3)	0.70 (0.50,0.83)	0.71 (0.52,0.87)
Exposure Year, N (%)		
2012	23 (10.7%)	23 (10.7%)
2013	12 (5.6%)	12 (5.6%)
2014	21 (9.8%)	21 (9.8%)
2015	20 (9.3%)	20 (9.3%)
2016	20 (9.3%)	20 (9.3%)
2017	59 (27.4%)	59 (27.4%)
2018	60 (27.9%)	60 (27.9%)

© 2023 Law AC et al. *JAMA Network Open.*

Table S8. Baseline patient characteristics of patients receiving MV ≥96 hours in sensitivity analysis, defining "closure-affected" hospitals as those discharging ≥30% of patients receiving a tracheostomy to a closing LTCH. Variables were included in multivariable adjustment. *Prior LTCH use = % of patients receiving tracheostomy discharged to any LTCH in the year prior to LTCH closure. **Social Vulnerability Index for each patient's census tract is based on 15 social factors, including poverty, lack of vehicle access, and crowded housing. A census tract's index is a percentile ranking and reflects the proportion of tracts in the country that are equal to or lower in terms of social vulnerability.

	Pre-period		Post-period	
**Characteristics**	**Closure-affected Hospital (N = 5525)**	**Matched Control Hospital (N = 5538)**	**Closure-affected Hospital (N = 5072)**	**Matched Control Hospital (N = 5437)**
Age, Mean±SD	76.2±7.2	76.2±7.3	75.8±7.1	76.4±7.3
Female, N (%)	2663 (48.2%)	2577 (46.5%)	2464 (48.6%)	2464 (45.3%)
Race, N (%)				
White non-Hispanic	4190 (75.8%)	4495 (81.2%)	3836 (75.6%)	4397 (80.9%)
Black non-Hispanic	1100 (19.9%)	676 (12.2%)	980 (19.3%)	643 (11.8%)
Other	235 (4.3%)	367 (6.6%)	256 (5.0%)	397 (7.3%)
Social vulnerability index, Mean±SD**	0.5±0.3	0.5±0.3	0.5±0.3	0.5±0.3
Medicaid Dual Eligibility, N (%)	1167 (21.1%)	1262 (22.8%)	1047 (20.6%)	1169 (21.5%)
Surgical patient, N (%)	2559 (46.3%)	2444 (44.1%)	2230 (44.0%)	2396 (44.1%)
Comorbidities, selected, N (%)				
Alzheimer's disease/dementia	1920 (34.8%)	1884 (34.0%)	1748 (34.5%)	1978 (36.4%)
Atrial fibrillation	2076 (37.6%)	2084 (37.6%)	1907 (37.6%)	2034 (37.4%)
Cancer	1038 (18.8%)	1032 (18.6%)	870 (17.2%)	1011 (18.6%)
Congestive heart failure	3884 (70.3%)	3732 (67.4%)	3465 (68.3%)	3728 (68.6%)
Chronic kidney disease	4327 (78.3%)	4340 (78.4%)	4085 (80.5%)	4425 (81.4%)
Chronic obstructive pulmonary disease and bronchiectasis	2870 (51.9%)	2721 (49.1%)	2564 (50.6%)	2626 (48.3%)
Depressive disorders	1954 (35.4%)	1944 (35.1%)	1862 (36.7%)	2038 (37.5%)
Diabetes	3040 (55.0%)	2933 (53.0%)	2761 (54.4%)	2949 (54.2%)
Hip/pelvic fracture	165 (3.0%)	205 (3.7%)	164 (3.2%)	194 (3.6%)
Hyperlipidemia	3794 (68.7%)	3749 (67.7%)	3533 (69.7%)	3822 (70.3%)
Hypertension	5110 (92.5%)	5068 (91.5%)	4707 (92.8%)	5031 (92.5%)
Ischemic heart disease	3999 (72.4%)	3931 (71.0%)	3535 (69.7%)	3863 (71.1%)
Stroke/transient ischemic attack	1496 (27.1%)	1547 (27.9%)	1431 (28.2%)	1544 (28.4%)
Acute organ dysfunction, N (%)				
Cardiovascular	3130 (56.7%)	3091 (55.8%)	2904 (57.3%)	3144 (57.8%)
Neurologic	2256 (40.8%)	2121 (38.3%)	2190 (43.2%)	2326 (42.8%)

© 2023 Law AC et al. *JAMA Network Open.*

Characteristics	Pre-period Closure-affected Hospital (N = 5525)	Pre-period Matched Control Hospital (N = 5538)	Post-period Closure-affected Hospital (N = 5072)	Post-period Matched Control Hospital (N = 5437)
Hematologic	1291 (23.4%)	1318 (23.8%)	1149 (22.7%)	1242 (22.8%)
Hepatic	407 (7.4%)	414 (7.5%)	461 (9.1%)	448 (8.2%)
Renal	3335 (60.4%)	3351 (60.5%)	3123 (61.6%)	3419 (62.9%)
Hospital size, N (%)				
Small (1 - 200)	466 (8.4%)	473 (8.5%)	366 (7.2%)	448 (8.2%)
Medium (201 - 400)	1950 (35.3%)	1594 (28.8%)	1694 (33.4%)	1459 (26.8%)
Large (> 400)	3109 (56.3%)	3471 (62.7%)	3012 (59.4%)	3530 (64.9%)
Ownership, N (%)				
For Profit	679 (12.3%)	561 (10.1%)	574 (11.3%)	510 (9.4%)
Private Nonprofit	4464 (80.8%)	4592 (82.9%)	4177 (82.4%)	4612 (84.8%)
Public	382 (6.9%)	385 (7.0%)	321 (6.3%)	315 (5.8%)
Teaching hospital, N (%)	4900 (88.7%)	4768 (86.1%)	4554 (89.8%)	4787 (88.0%)
Safety net hospital, N (%)	1397 (25.3%)	1419 (25.6%)	1307 (25.8%)	1468 (27.0%)
Hospital Region, N (%)				
Northeast	505 (9.1%)	932 (16.8%)	546 (10.8%)	937 (17.2%)
Midwest	917 (16.6%)	731 (13.2%)	860 (17.0%)	745 (13.7%)
South	3597 (65.1%)	3216 (58.1%)	3162 (62.3%)	3069 (56.4%)
West	506 (9.2%)	659 (11.9%)	504 (9.9%)	686 (12.6%)
Prior LTCH use*, Median (IQR), N (%)	0.66 (0.50,0.80)	0.69 (0.47,0.80)	0.64 (0.46,0.80)	0.71 (0.47,0.79)
Rural, N (%)	27 (0.5%)	43 (0.8%)	23 (0.5%)	54 (1.0%)
Year, N (%)				
2011	650 (11.8%)	702 (12.7%)	0 (0.0%)	0 (0.0%)
2012	371 (6.7%)	271 (4.9%)	0 (0.0%)	0 (0.0%)
2013	386 (7.0%)	408 (7.4%)	679 (13.4%)	708 (13.0%)
2014	341 (6.2%)	273 (4.9%)	264 (5.2%)	239 (4.4%)
2015	659 (11.9%)	592 (10.7%)	383 (7.6%)	479 (8.8%)
2016	1359 (24.6%)	1047 (18.9%)	415 (8.2%)	277 (5.1%)
2017	1759 (31.8%)	2245 (40.5%)	541 (10.7%)	556 (10.2%)
2018	0 (0.0%)	0 (0.0%)	1173 (23.1%)	992 (18.2%)
2019	0 (0.0%)	0 (0.0%)	1617 (31.9%)	2186 (40.2%)

© 2023 Law AC et al. *JAMA Network Open.*

Table S9. Baseline patient characteristics of patients receiving a tracheostomy in sensitivity analysis, defining "closure-affected" hospitals as those discharging :=30% of patients receiving a tracheostomy to a closing LTCH. Variables were included in multivariable adjustment.
*Prior LTCH use = % of patients receiving tracheostomy discharged to any LTCH in the year prior to LTCH closure.
**Social Vulnerability Index for each patient's census tract is based on 15 social factors, including poverty, lack of vehicle access, and crowded housing. A census tract's index is a percentile ranking and reflects the proportion of tracts in the country that are equal to or lower in terms of social vulnerability.

	Pre-period		Post-period	
**Characteristics**	**Closure-affected Hospital (N = 1113)**	**Matched Control Hospital (N = 1152)**	**Closure-affected Hospital (N = 961)**	**Matched Control Hospital (N = 1108)**
Age, Mean±SD	75.1±6.6	75.2±6.8	74.7±6.4	75.3±6.6
Female, N (%)	548 (49.2%)	529 (45.9%)	479 (49.8%)	470 (42.4%)
Race, N (%)				
White non-Hispanic	833 (74.8%)	920 (79.9%)	717 (74.6%)	874 (78.9%)
Black non-Hispanic	239 (21.5%)	154 (13.4%)	191 (19.9%)	150 (13.5%)
Other	41 (3.7%)	78 (6.8%)	53 (5.5%)	84 (7.6%)
Social vulnerability index, Mean±SD**	0.5±0.3	0.5±0.3	0.5±0.3	0.5±0.3
Medicaid Dual Eligibility, N (%)	215 (19.3%)	240 (20.8%)	183 (19.0%)	214 (19.3%)
Surgical patient, N (%)	1014 (91.1%)	1001 (86.9%)	814 (84.7%)	936 (84.5%)
Comorbidities, selected, N (%)				
Alzheimer's disease/dementia	362 (32.5%)	380 (33.0%)	301 (31.3%)	410 (37.0%)
Atrial fibrillation	445 (40.0%)	485 (42.1%)	405 (42.1%)	434 (39.2%)
Cancer	198 (17.8%)	204 (17.7%)	153 (15.9%)	206 (18.6%)
Congestive heart failure	778 (69.9%)	791 (68.7%)	662 (68.9%)	727 (65.6%)
Chronic kidney disease	865 (77.7%)	884 (76.7%)	766 (79.7%)	873 (78.8%)
Chronic obstructive pulmonary disease and bronchiectasis	602 (54.1%)	586 (50.9%)	521 (54.2%)	554 (50.0%)
Depressive disorders	396 (35.6%)	448 (38.9%)	360 (37.5%)	430 (38.8%)
Diabetes	640 (57.5%)	635 (55.1%)	553 (57.5%)	612 (55.2%)
Hip/pelvic fracture	25 (2.2%)	36 (3.1%)	28 (2.9%)	41 (3.7%)
Hyperlipidemia	761 (68.4%)	788 (68.4%)	684 (71.2%)	781 (70.5%)
Hypertension	1038 (93.3%)	1064 (92.4%)	904 (94.1%)	1030 (93.0%)
Ischemic heart disease	785 (70.5%)	785 (68.1%)	677 (70.4%)	758 (68.4%)
Stroke/transient ischemic attack	371 (33.3%)	376 (32.6%)	337 (35.1%)	382 (34.5%)
Acute organ dysfunction, N (%)				
Cardiovascular	599 (53.8%)	601 (52.2%)	509 (53.0%)	572 (51.6%)
Neurologic	471 (42.3%)	404 (35.1%)	399 (41.5%)	467 (42.1%)
Hematologic	259 (23.3%)	245 (21.3%)	209 (21.7%)	255 (23.0%)

© 2023 Law AC et al. *JAMA Network Open.*

Characteristics	Pre-period		Post-period	
	Closure-affected Hospital (N = 1113)	Matched Control Hospital (N = 1152)	Closure-affected Hospital (N = 961)	Matched Control Hospital (N = 1108)
Hepatic	78 (7.0%)	62 (5.4%)	70 (7.3%)	68 (6.1%)
Renal	649 (58.3%)	655 (56.9%)	569 (59.2%)	629 (56.8%)
Hospital size, N (%)				
Small (1 - 200)	76 (6.8%)	76 (6.6%)	52 (5.4%)	61 (5.5%)
Medium (201 - 400)	354 (31.8%)	285 (24.7%)	310 (32.3%)	218 (19.7%)
Large (> 400)	683 (61.4%)	791 (68.7%)	599 (62.3%)	829 (74.8%)
Ownership, N (%)				
For Profit	139 (12.5%)	116 (10.1%)	126 (13.1%)	92 (8.3%)
Private Nonprofit	922 (82.8%)	977 (84.8%)	775 (80.6%)	958 (86.5%)
Public	52 (4.7%)	59 (5.1%)	60 (6.2%)	58 (5.2%)
Teaching hospital, N (%)	1014 (91.1%)	1020 (88.5%)	888 (92.4%)	998 (90.1%)
Safety net hospital, N (%)	317 (28.5%)	308 (26.7%)	278 (28.9%)	317 (28.6%)
Hospital Region, N (%)				
Northeast	116 (10.4%)	190 (16.5%)	114 (11.9%)	229 (20.7%)
Midwest	212 (19.0%)	139 (12.1%)	173 (18.0%)	163 (14.7%)
South	702 (63.1%)	670 (58.2%)	571 (59.4%)	588 (53.1%)
West	83 (7.5%)	153 (13.3%)	103 (10.7%)	128 (11.6%)
Prior LTCH use*, Median (IQR), N (%)	0.61 (0.46,0.80)	0.69 (0.46,0.77)	0.60 (0.45,0.78)	0.69 (0.46,0.77)
Rural, N (%)	3 (0.3%)	6 (0.5%)	1 (0.1%)	5 (0.5%)
Year, N (%)				
2011	148 (13.3%)	157 (13.6%)	0 (0.0%)	0 (0.0%)
2012	47 (4.2%)	56 (4.9%)	0 (0.0%)	0 (0.0%)
2013	86 (7.7%)	75 (6.5%)	143 (14.9%)	148 (13.4%)
2014	88 (7.9%)	50 (4.3%)	44 (4.6%)	34 (3.1%)
2015	147 (13.2%)	121 (10.5%)	72 (7.5%)	122 (11.0%)
2016	278 (25.0%)	185 (16.1%)	107 (11.1%)	47 (4.2%)
2017	319 (28.7%)	508 (44.1%)	106 (11.0%)	107 (9.7%)
2018	0 (0.0%)	0 (0.0%)	224 (23.3%)	176 (15.9%)
2019	0 (0.0%)	0 (0.0%)	265 (27.6%)	474 (42.8%)

© 2023 Law AC et al. *JAMA Network Open.*

Table S10. Baseline patient characteristics of patients receiving MV ≥96 hours in sensitivity analysis, defining "closure-affected" hospitals as those discharging ≥0% of patients receiving a tracheostomy to a closing LTCH. Variables were included in multivariable adjustment. *Prior LTCH use = % of patients receiving tracheostomy discharged to any LTCH in the year prior to LTCH closure. **Social Vulnerability Index for each patient's census tract is based on 15 social factors, including poverty, lack of vehicle access, and crowded housing. A census tract's index is a percentile ranking and reflects the proportion of tracts in the country that are equal to or lower in terms of social vulnerability.

	**Pre-period**		**Post-period**	
**Characteristics**	**Closure-affected Hospital (N = 14606)**	**Matched Control Hospital (N = 12730)**	**Closure-affected Hospital (N = 13985)**	**Matched Control Hospital (N = 12359)**
Age, Mean±SD	76.0±7.2	76.3±7.3	75.7±7.1	76.2±7.3
Female, N (%)	6912 (47.3%)	5934 (46.6%)	6534 (46.7%)	5622 (45.5%)
Race, N (%)				
White non-Hispanic	11329 (77.6%)	10269 (80.7%)	10755 (76.9%)	9811 (79.4%)
Black non-Hispanic	2606 (17.8%)	1723 (13.5%)	2483 (17.8%)	1758 (14.2%)
Other	671 (4.6%)	738 (5.8%)	747 (5.3%)	790 (6.4%)
Social vulnerability index, Mean±SD**	0.5±0.3	0.5±0.3	0.5±0.3	0.5±0.3
Medicaid Dual Eligibility, N (%)	3018 (20.7%)	2819 (22.1%)	2889 (20.7%)	2690 (21.8%)
Surgical patient, N (%)	7199 (49.3%)	5934 (46.6%)	6542 (46.8%)	5488 (44.4%)
Comorbidities, selected, N (%)				
Alzheimer's disease/dementia	4875 (33.4%)	4249 (33.4%)	4831 (34.5%)	4420 (35.8%)
Atrial fibrillation	5380 (36.8%)	4812 (37.8%)	5128 (36.7%)	4592 (37.2%)
Cancer	2606 (17.8%)	2368 (18.6%)	2424 (17.3%)	2223 (18.0%)
Congestive heart failure	9947 (68.1%)	8729 (68.6%)	9373 (67.0%)	8561 (69.3%)
Chronic kidney disease	11342 (77.7%)	9918 (77.9%)	11193 (80.0%)	9946 (80.5%)
Chronic obstructive pulmonary disease and bronchiectasis	7310 (50.0%)	6422 (50.4%)	6797 (48.6%)	6106 (49.4%)
Depressive disorders	5160 (35.3%)	4448 (34.9%)	5143 (36.8%)	4465 (36.1%)
Diabetes	7933 (54.3%)	6842 (53.7%)	7470 (53.4%)	6642 (53.7%)
Hip/pelvic fracture	495 (3.4%)	460 (3.6%)	481 (3.4%)	424 (3.4%)
Hyperlipidemia	9846 (67.4%)	8629 (67.8%)	9668 (69.1%)	8592 (69.5%)
Hypertension	13511 (92.5%)	11738 (92.2%)	12871 (92.0%)	11354 (91.9%)
Ischemic heart disease	10429 (71.4%)	9146 (71.8%)	9734 (69.6%)	8789 (71.1%)
Stroke/transient ischemic attack	4273 (29.3%)	3576 (28.1%)	4262 (30.5%)	3538 (28.6%)
Acute organ dysfunction, N (%)				
Cardiovascular	7969 (54.6%)	6921 (54.4%)	7945 (56.8%)	6983 (56.5%)
Neurologic	5888 (40.3%)	4747 (37.3%)	6078 (43.5%)	5484 (44.4%)
Hematologic	3398 (23.3%)	2975 (23.4%)	3239 (23.2%)	2725 (22.0%)
Hepatic	1049 (7.2%)	1006 (7.9%)	1145 (8.2%)	1007 (8.1%)

© 2023 Law AC et al. *JAMA Network Open.*

	Pre-period		Post-period	
**Characteristics**	**Closure-affected Hospital (N = 14606)**	**Matched Control Hospital (N = 12730)**	**Closure-affected Hospital (N = 13985)**	**Matched Control Hospital (N = 12359)**
Renal	8671 (59.4%)	7661 (60.2%)	8550 (61.1%)	7729 (62.5%)
Hospital size, N (%)				
Small (1 - 200)	777 (5.3%)	787 (6.2%)	682 (4.9%)	733 (5.9%)
Medium (201 - 400)	3494 (23.9%)	3527 (27.7%)	3195 (22.8%)	3341 (27.0%)
Large (> 400)	10335 (70.8%)	8416 (66.1%)	10108 (72.3%)	8285 (67.0%)
Ownership, N (%)				
For Profit	1653 (11.3%)	1396 (11.0%)	1431 (10.2%)	1298 (10.5%)
Private Nonprofit	11421 (78.2%)	10030 (78.8%)	11008 (78.7%)	9893 (80.0%)
Public	1532 (10.5%)	1304 (10.2%)	1546 (11.1%)	1168 (9.5%)
Teaching hospital, N (%)	13283 (90.9%)	11323 (88.9%)	12820 (91.7%)	11135 (90.1%)
Safety net hospital, N (%)	5013 (34.3%)	4728 (37.1%)	4865 (34.8%)	4588 (37.1%)
Hospital Region, N (%)				
Northeast	2380 (16.3%)	2339 (18.4%)	2446 (17.5%)	2333 (18.9%)
Midwest	2667 (18.3%)	2238 (17.6%)	2685 (19.2%)	2156 (17.4%)
South	8543 (58.5%)	7103 (55.8%)	7874 (56.3%)	6772 (54.8%)
West	1016 (7.0%)	1050 (8.2%)	980 (7.0%)	1098 (8.9%)
Prior LTCH use*, Median (IQR), N (%)	0.67 (0.50,0.80)	0.69 (0.50,0.80)	0.67 (0.50,0.80)	0.69 (0.50,0.80)
Rural, N (%)	27 (0.2%)	43 (0.3%)	23 (0.2%)	54 (0.4%)
Year, N (%)				
2011	1797 (12.3%)	1450 (11.4%)	0 (0.0%)	0 (0.0%)
2012	537 (3.7%)	384 (3.0%)	0 (0.0%)	0 (0.0%)
2013	1756 (12.0%)	1460 (11.5%)	1936 (13.8%)	1433 (11.6%)
2014	1754 (12.0%)	1332 (10.5%)	421 (3.0%)	342 (2.8%)
2015	1306 (8.9%)	1144 (9.0%)	1796 (12.8%)	1598 (12.9%)
2016	3984 (27.3%)	3135 (24.6%)	1857 (13.3%)	1195 (9.7%)
2017	3472 (23.8%)	3825 (30.0%)	1067 (7.6%)	1067 (8.6%)
2018	0 (0.0%)	0 (0.0%)	3741 (26.8%)	3065 (24.8%)
2019	0 (0.0%)	0 (0.0%)	3167 (22.6%)	3659 (29.6%)

© 2023 Law AC et al. *JAMA Network Open.*

Table S11. Baseline patient characteristics of patients receiving a tracheostomy in sensitivity analysis, defining “closure-affected” hospitals as those discharging :=0% of patients receiving a tracheostomy to a closing LTCH. Variables were included in multivariable adjustment. *Prior LTCH use = % of patients receiving tracheostomy discharged to any LTCH in the year prior to LTCH closure. **Social Vulnerability Index for each patient’s census tract is based on 15 social factors, including poverty, lack of vehicle access, and crowded housing. A census tract’s index is a percentile ranking and reflects the proportion of tracts in the country that are equal to or lower in terms of social vulnerability.

	**Pre-period**		**Post-period**	
**Characteristics**	**Closure-affected Hospital (N = 3408)**	**Matched Control Hospital (N = 2673)**	**Closure-affected Hospital (N = 3032)**	**Matched Control Hospital (N = 2525)**
Age, Mean±SD	74.9±6.5	75.4±6.9	74.7±6.4	75.2±6.7
Female, N (%)	1610 (47.2%)	1197 (44.8%)	1410 (46.5%)	1108 (43.9%)
Race, N (%)				
White non-Hispanic	2611 (76.6%)	2095 (78.4%)	2318 (76.5%)	1954 (77.4%)
Black non-Hispanic	637 (18.7%)	420 (15.7%)	541 (17.8%)	403 (16.0%)
Other	160 (4.7%)	158 (5.9%)	173 (5.7%)	168 (6.7%)
Social vulnerability index, Mean±SD**	0.5±0.3	0.5±0.3	0.5±0.3	0.5±0.3
Medicaid Dual Eligibility, N (%)	624 (18.3%)	557 (20.8%)	579 (19.1%)	510 (20.2%)
Surgical patient, N (%)	3094 (90.8%)	2400 (89.8%)	2631 (86.8%)	2155 (85.3%)
Comorbidities, selected, N (%)				
Alzheimer's disease/dementia	1043 (30.6%)	878 (32.8%)	1019 (33.6%)	920 (36.4%)
Atrial fibrillation	1370 (40.2%)	1122 (42.0%)	1257 (41.5%)	1005 (39.8%)
Cancer	581 (17.0%)	505 (18.9%)	482 (15.9%)	455 (18.0%)
Congestive heart failure	2332 (68.4%)	1830 (68.5%)	2042 (67.3%)	1704 (67.5%)
Chronic kidney disease	2621 (76.9%)	2022 (75.6%)	2398 (79.1%)	1988 (78.7%)
Chronic obstructive pulmonary disease and bronchiectasis	1783 (52.3%)	1404 (52.5%)	1575 (51.9%)	1282 (50.8%)
Depressive disorders	1240 (36.4%)	1016 (38.0%)	1160 (38.3%)	966 (38.3%)
Diabetes	1901 (55.8%)	1486 (55.6%)	1684 (55.5%)	1400 (55.4%)
Hip/pelvic fracture	112 (3.3%)	104 (3.9%)	111 (3.7%)	93 (3.7%)
Hyperlipidemia	2316 (68.0%)	1785 (66.8%)	2086 (68.8%)	1775 (70.3%)
Hypertension	3186 (93.5%)	2478 (92.7%)	2826 (93.2%)	2344 (92.8%)
Ischemic heart disease	2390 (70.1%)	1845 (69.0%)	2080 (68.6%)	1758 (69.6%)
Stroke/transient ischemic attack	1194 (35.0%)	909 (34.0%)	1137 (37.5%)	868 (34.4%)
Acute organ dysfunction, N (%)				
Cardiovascular	1719 (50.4%)	1339 (50.1%)	1592 (52.5%)	1289 (51.0%)
Neurologic	1384 (40.6%)	975 (36.5%)	1304 (43.0%)	1079 (42.7%)
Hematologic	766 (22.5%)	554 (20.7%)	662 (21.8%)	530 (21.0%)
Hepatic	208 (6.1%)	150 (5.6%)	193 (6.4%)	163 (6.5%)

© 2023 Law AC et al. *JAMA Network Open.*

Characteristics	Pre-period Closure-affected Hospital (N = 3408)	Pre-period Matched Control Hospital (N = 2673)	Post-period Closure-affected Hospital (N = 3032)	Post-period Matched Control Hospital (N = 2525)
Renal	1932 (56.7%)	1504 (56.3%)	1745 (57.6%)	1458 (57.7%)
Hospital size, N (%)				
Small (1 - 200)	159 (4.7%)	131 (4.9%)	117 (3.9%)	134 (5.3%)
Medium (201 - 400)	765 (22.4%)	602 (22.5%)	632 (20.8%)	530 (21.0%)
Large (> 400)	2484 (72.9%)	1940 (72.6%)	2283 (75.3%)	1861 (73.7%)
Ownership, N (%)				
For Profit	364 (10.7%)	269 (10.1%)	331 (10.9%)	244 (9.7%)
Private Nonprofit	2675 (78.5%)	2118 (79.2%)	2360 (77.8%)	2003 (79.3%)
Public	369 (10.8%)	286 (10.7%)	341 (11.2%)	278 (11.0%)
Teaching hospital, N (%)	3142 (92.2%)	2435 (91.1%)	2844 (93.8%)	2320 (91.9%)
Safety net hospital, N (%)	1263 (37.1%)	1104 (41.3%)	1179 (38.9%)	1044 (41.3%)
Hospital Region, N (%)				
Northeast	627 (18.4%)	503 (18.8%)	623 (20.5%)	506 (20.0%)
Midwest	666 (19.5%)	434 (16.2%)	623 (20.5%)	423 (16.8%)
South	1931 (56.7%)	1524 (57.0%)	1602 (52.8%)	1408 (55.8%)
West	184 (5.4%)	212 (7.9%)	184 (6.1%)	188 (7.4%)
Prior LTCH use*, Median (IQR), N (%)	0.66 (0.48,0.79)	0.66 (0.46,0.76)	0.64 (0.47,0.79)	0.67 (0.46,0.77)
Rural, N (%)	3 (0.1%)	6 (0.2%)	1 (0.0%)	5 (0.2%)
Year, N (%)				
2011	436 (12.8%)	314 (11.7%)	0 (0.0%)	0 (0.0%)
2012	77 (2.3%)	77 (2.9%)	0 (0.0%)	0 (0.0%)
2013	457 (13.4%)	297 (11.1%)	446 (14.7%)	281 (11.1%)
2014	517 (15.2%)	306 (11.4%)	68 (2.2%)	61 (2.4%)
2015	300 (8.8%)	240 (9.0%)	431 (14.2%)	337 (13.3%)
2016	927 (27.2%)	624 (23.3%)	496 (16.4%)	283 (11.2%)
2017	694 (20.4%)	815 (30.5%)	226 (7.5%)	188 (7.4%)
2018	0 (0.0%)	0 (0.0%)	751 (24.8%)	608 (24.1%)
2019	0 (0.0%)	0 (0.0%)	614 (20.3%)	767 (30.4%)

© 2023 Law AC et al. *JAMA Network Open.*

Table S12. Unadjusted outcomes in sensitivity analysis, defining “closure-affected” hospitals as those discharging :=30% of patients receiving a tracheostomy to a closing LTCH. Unadjusted outcomes of patients receiving MV :=96 hours (A) and Tracheostomy (B) at Closure-affected and matched Control hospitals.

© 2023 Law AC et al. *JAMA Network Open.*

A. Patients receiving MV ≥96 hours				
	**Pre-period**		**Post-period**	
**Outcomes**	**Closure-affected Hospital (N = 5525)**	**Matched Control Hospital (N = 5538)**	**Closure-affected Hospital (N = 5072)**	**Matched Control Hospital (N = 5437)**
90-day mortality, N (%)	3341 (60.5%)	3363 (60.7%)	3105 (61.2%)	3335 (61.3%)
Tracheostomy, N (%)	1113 (20.1%)	1152 (20.8%)	961 (18.9%)	1108 (20.4%)
Palliative care delivery, N (%)	1361 (24.6%)	1458 (26.3%)	1421 (28.0%)	1587 (29.2%)
DNR, N (%)	1538 (27.8%)	1546 (27.9%)	1551 (30.6%)	1745 (32.1%)
IFD in 90, days				
Mean±SD	12.7±22.9	12.6±22.8	14.6±23.8	13.9±23.4
Median (IQR)	0.0 (0.0,13.0)	0.0 (0.0,14.0)	0.0 (0.0,24.0)	0.0 (0.0,21.0)
Spending/days alive in 90 days, $				
Mean±SD	87532.69±70748.09 (5525)	91155.09±88266.41 (5538)	89718.90±73797.57 (5072)	96594.66±87500.69 (5437)
Median (IQR)	62703.83 (37824.00,118682.22)	61301.08 (37206.00,122128.36)	64596.00 (38339.50,121163.00)	65191.34 (40168.00,128205.72)
Index hospital length of stay, days				
Mean±SD	18.2±12.3	18.4±13.7	18.0±12.1	18.8±14.7
Median (IQR)	15.0 (10.0,22.0)	15.0 (10.0,22.0)	15.0 (10.0,22.0)	15.0 (10.0,23.0)
Hospital disposition, N (%)				
LTCH	1122 (20.3%)	1059 (19.1%)	825 (16.3%)	1047 (19.3%)
SNF or IRF	1039 (18.8%)	1154 (20.8%)	1024 (20.2%)	1113 (20.5%)
Hospice (facility or home)	637 (11.5%)	559 (10.1%)	568 (11.2%)	568 (10.4%)
Home	321 (5.8%)	338 (6.1%)	271 (5.3%)	262 (4.8%)
Death	2107 (38.1%)	2176 (39.3%)	2088 (41.2%)	2270 (41.8%)
**B. Subgroup receiving Tracheostomy**				
	**Pre-period**		**Post-period**	
	**Closure-affected Hospital (N = 1113)**	**Matched Control Hospital (N = 1152)**	**Closure-affected Hospital (N = 961)**	**Matched Control Hospital (N = 1108)**
90-day mortality, N (%)	477 (42.9%)	483 (41.9%)	407 (42.4%)	460 (41.5%)
Palliative care delivery, N (%)	124 (11.1%)	146 (12.7%)	136 (14.2%)	157 (14.2%)
DNR, N (%)	109 (9.8%)	158 (13.7%)	136 (14.2%)	170 (15.3%)
IFD in 90, days				
Mean±SD	7.4±15.7	8.0±16.5	9.4±17.7	9.5±18.6
Median (IQR)	0.0 (0.0,4.0)	0.0 (0.0,4.0)	0.0 (0.0,11.0)	0.0 (0.0,7.0)
Spending/days alive in 90 days, $				

© 2023 Law AC et al. *JAMA Network Open.*

Mean±SD	176909.11±76785.30 (1113)	188988.14±120374.93 (1152)	180631.67±76703.30 (961)	198510.69±103824.65 (1108)
Median (IQR)	168963.76 (125194.00,214744.56	168983.91 (128092.19,218037.89	173849.80 (127627.00,221203.42	182543.09 (134567.83,231677.59
Index hospital length of stay, days				
Mean±SD	28.0±16.7	27.6±19.0	27.9±16.4	28.7±19.4
Median (IQR)	24.0 (18.0,33.0)	22.0 (16.0,33.0)	24.0 (18.0,32.0)	24.0 (17.0,34.0)
Hospital disposition, N (%)				
LTCH	673 (60.5%)	713 (61.9%)	513 (53.4%)	703 (63.4%)
SNF or IRF	106 (9.5%)	109 (9.5%)	131 (13.6%)	105 (9.5%)
Hospice (facility or home)	46 (4.1%)	42 (3.6%)	38 (4.0%)	50 (4.5%)
Home	21 (1.9%)	26 (2.3%)	24 (2.5%)	25 (2.3%)
Death	196 (17.6%)	206 (17.9%)	174 (18.1%)	201 (18.1%)

© 2023 Law AC et al. *JAMA Network Open.*

Table S13. Unadjusted outcomes in sensitivity analysis, defining “closure-affected” hospitals as those discharging :=0% of patients receiving a tracheostomy to a closing LTCH. Unadjusted outcomes of patients receiving MV :=96 hours (A) and Tracheostomy (B) at Closure-affected and matched Control hospitals.

© 2023 Law AC et al. *JAMA Network Open.*

A. Patients receiving MV ≥96 hours				
	Pre-period		Post-period	
**Outcomes**	**Closure-affected Hospital (N = 14606)**	**Matched Control Hospital (N = 12730)**	**Closure-affected Hospital (N = 13985)**	**Matched Control Hospital (N = 12359)**
90-day mortality, N (%)	8780 (60.1%)	7700 (60.5%)	8418 (60.2%)	7554 (61.1%)
Tracheostomy, N (%)	3408 (23.3%)	2673 (21.0%)	3032 (21.7%)	2525 (20.4%)
Palliative care delivery, N (%)	3769 (25.8%)	3139 (24.7%)	4029 (28.8%)	3513 (28.4%)
DNR, N (%)	3807 (26.1%)	3470 (27.3%)	4346 (31.1%)	3967 (32.1%)
IFD in 90, days				
Mean±SD	11.9±22.1	12.3±22.6	13.6±23.2	13.7±23.5
Median (IQR)	0.0 (0.0,11.0)	0.0 (0.0,12.0)	0.0 (0.0,20.0)	0.0 (0.0,20.0)
Spending/days alive in 90 days, $				
Mean±SD	91802.17±75856.88 (14606)	89886.65±80039.33 (12730)	93829.28±77335.31 (13985)	92754.99±83075.59 (12359)
Median (IQR)	66415.67 (39198.00,127875.00)	62227.89 (37632.00,120590.91)	66371.76 (39809.00,129182.41)	63141.26 (38836.00,124670.00)
Index hospital length of stay, days				
Mean±SD	19.1±13.3	18.8±13.7	18.8±13.2	18.7±14.0
Median (IQR)	16.0 (11.0,24.0)	15.0 (10.0,23.0)	16.0 (10.0,23.0)	15.0 (10.0,23.0)
Hospital disposition, N (%)				
LTCH	3327 (22.8%)	2514 (19.7%)	2837 (20.3%)	2381 (19.3%)
SNF or IRF	2730 (18.7%)	2658 (20.9%)	2693 (19.3%)	2537 (20.5%)
Hospice (facility or home)	1430 (9.8%)	1291 (10.1%)	1469 (10.5%)	1332 (10.8%)
Home	780 (5.3%)	717 (5.6%)	712 (5.1%)	605 (4.9%)
Death	5677 (38.9%)	4957 (38.9%)	5597 (40.0%)	5081 (41.1%)
**B. Subgroup receiving Tracheostomy**				
	**Pre-period**		**Post-period**	
	**Closure-affected Hospital (N = 3408)**	**Matched Control Hospital (N = 2673)**	**Closure-affected Hospital (N = 3032)**	**Matched Control Hospital (N = 2525)**
90-day mortality, N (%)	1475 (43.3%)	1156 (43.2%)	1258 (41.5%)	1063 (42.1%)
Palliative care delivery, N (%)	405 (11.9%)	304 (11.4%)	394 (13.0%)	328 (13.0%)
DNR, N (%)	356 (10.4%)	342 (12.8%)	409 (13.5%)	368 (14.6%)
IFD in 90, days				
Mean±SD	7.4±15.9	7.3±16.0	8.5±17.2	9.0±18.1
Median (IQR)	0.0 (0.0,4.0)	0.0 (0.0,2.0)	0.0 (0.0,6.0)	0.0 (0.0,6.0)
Spending/days alive in 90 days, $				

© 2023 Law AC et al. *JAMA Network Open.*

Mean±SD	176480.43±77042.40 (3408)	181775.17±97877.61 (2673)	185410.94±77709.91 (3032)	188969.72±97924.48 (2525)
Median (IQR)	169271.26 (127245.16,212923.42	169137.15 (127182.76,214871.50	177872.03 (133807.18,224939.88	175547.10 (129972.50,221648.32
Index hospital length of stay, days				
Mean±SD	28.4±17.6	28.4±18.8	28.2±17.9	28.5±19.2
Median (IQR)	24.0 (17.0,34.0)	23.0 (17.0,34.0)	24.0 (17.0,33.0)	24.0 (17.0,34.0)
Hospital disposition, N (%)				
LTCH	2151 (63.1%)	1620 (60.6%)	1836 (60.6%)	1567 (62.1%)
SNF or IRF	295 (8.7%)	296 (11.1%)	330 (10.9%)	265 (10.5%)
Hospice (facility or home)	114 (3.3%)	86 (3.2%)	104 (3.4%)	93 (3.7%)
Home	64 (1.9%)	51 (1.9%)	54 (1.8%)	51 (2.0%)
Death	642 (18.8%)	486 (18.2%)	534 (17.6%)	467 (18.5%)

© 2023 Law AC et al. *JAMA Network Open.*

Figure S1. Sensitivity analysis with hospital of admission as fixed effect, among patients receiving mechanical ventilation :=96 hours. Estimates (95% confidence intervals) of absolute change in outcomes generated by difference-in-differences analysis adjusted for patient-level characteristics, with hospital of admission as a random effect, are shown. DNR = do-not-resuscitate; LOS = length of stay; LTCH = long-term acute care hospital; SNF = skilled nursing facility; IFD = alive-and-institution-free days.

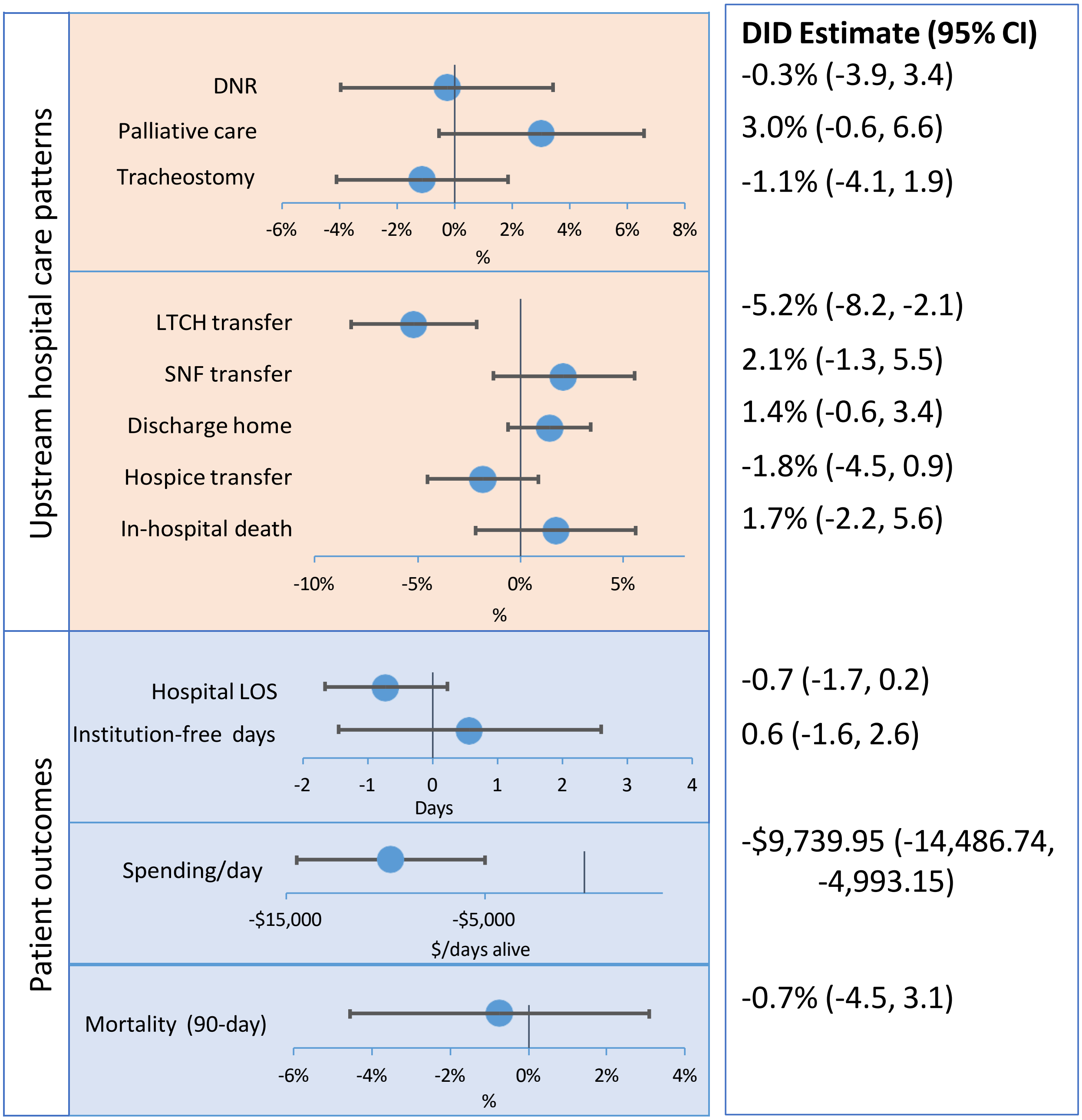

© 2023 Law AC et al. *JAMA Network Open.*

Figure S2. Sensitivity analysis with hospital of admission as fixed effect, among patients receiving a tracheostomy. Estimates (95% confidence intervals) of absolute change in outcomes generated by difference-in-differences analysis adjusted for patient-level characteristics, with hospital of admission as a random effect, are shown. DNR = do-not-resuscitate; LOS = length of stay; LTCH = long-term acute care hospital; SNF = skilled nursing facility; IFD = alive-and-institution-free days.

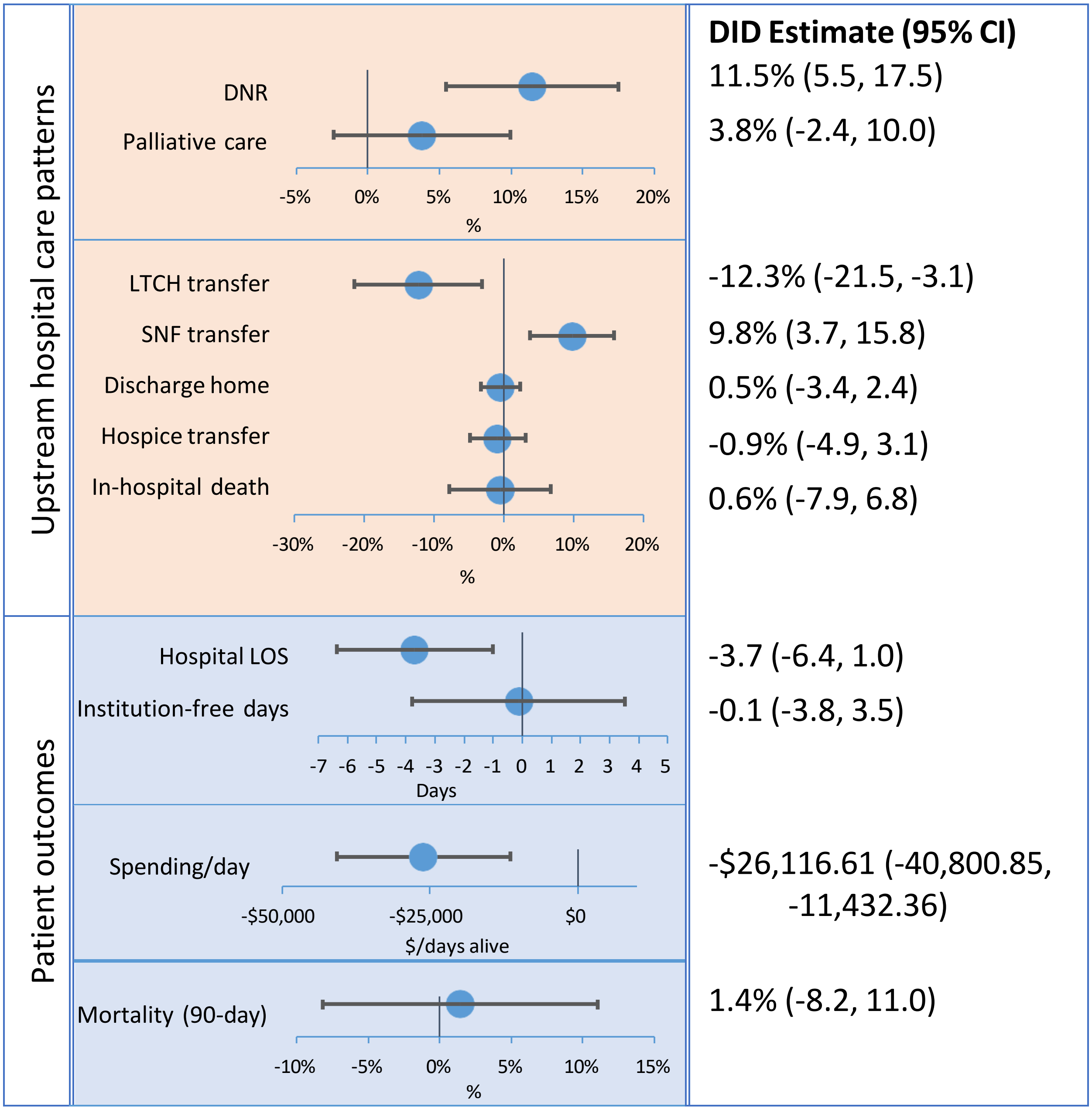

© 2023 Law AC et al. *JAMA Network Open.*

Figure S3. Adjusted outcomes in sensitivity analysis varying definition of “closure-affected” hospital, among patients receiving MV :=96 hours. The threshold to define a “closure-affected” hospital is varied as shown. Estimates (95% confidence intervals) of change in outcomes generated by difference-in-differences analysis adjusted for hospital- and patient-level characteristics, with matched exposure-control hospital pairs as random effect, are shown. DNR = do-not-resuscitate; LOS = length of stay; LTCH = long-term acute care hospital; SNF = skilled nursing facility; IFD = alive-and-institution-free days.

© 2023 Law AC et al. *JAMA Network Open.*

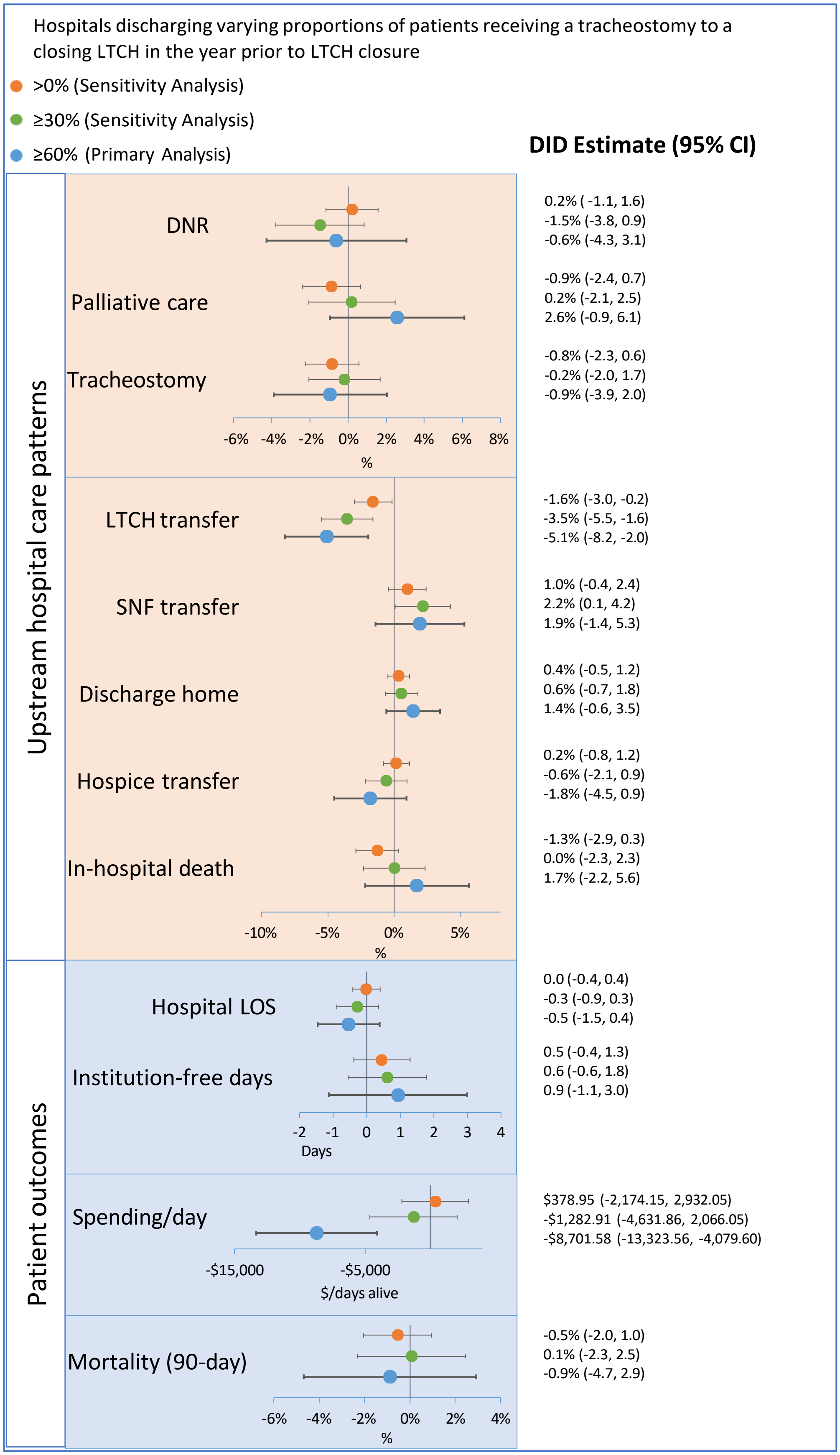

© 2023 Law AC et al. *JAMA Network Open.*

Figure S4. Adjusted outcomes in sensitivity analysis varying definition of "closure-affected" hospital, among patients receiving a tracheostomy. The threshold to define a "closure-affected" hospital is varied as shown. Estimates (95% confidence intervals) of change in outcomes generated by difference-in-differences analysis adjusted for hospital- and patient-level characteristics, with matched exposure-control hospital pairs as random effect, are shown. DNR = do-not-resuscitate; LOS = length of stay; LTCH = long-term acute care hospital; SNF = skilled nursing facility; IFD = alive-and-institution-free days.

© 2023 Law AC et al. *JAMA Network Open.*

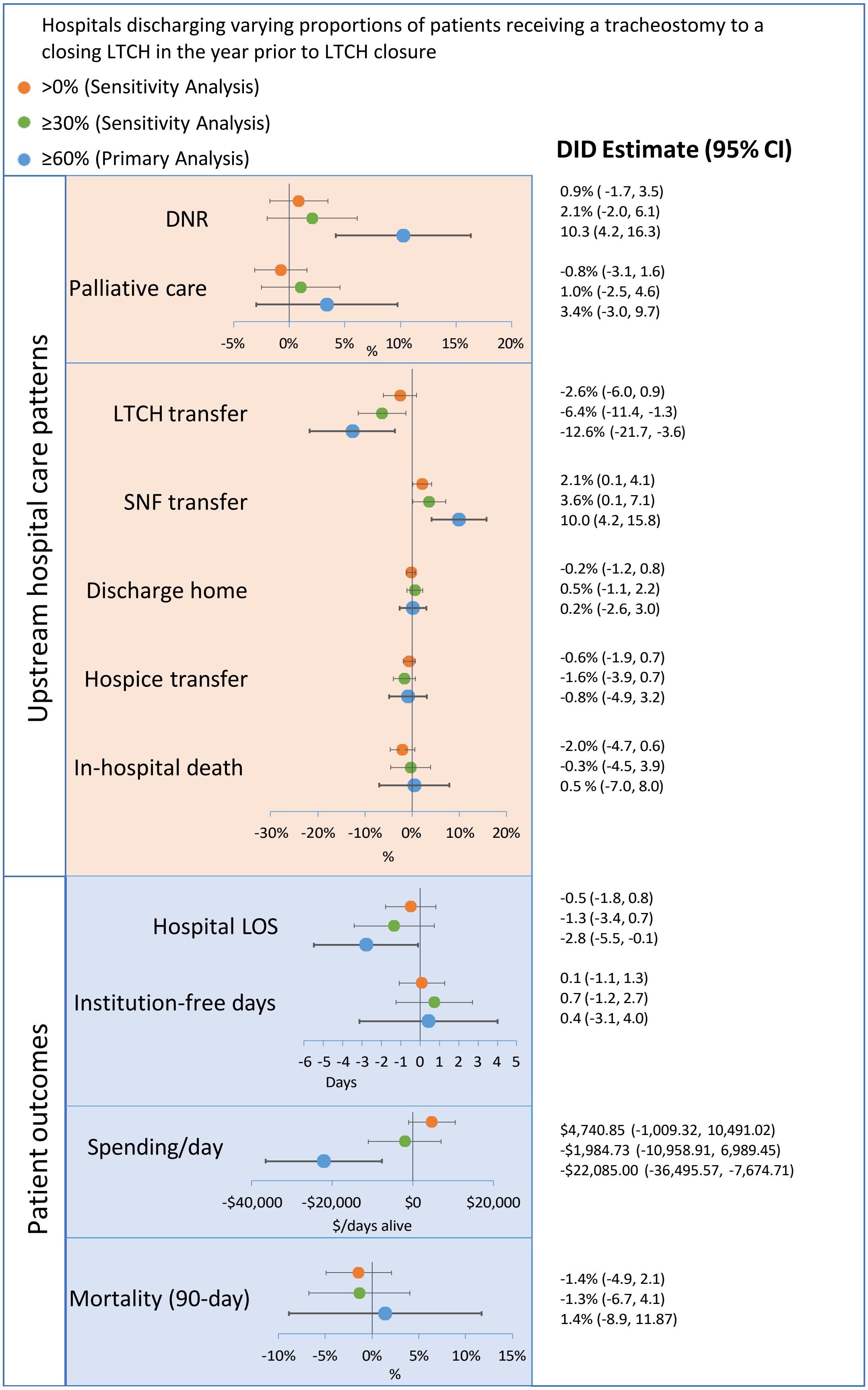

© 2023 Law AC et al. *JAMA Network Open.*

Figure S5. Adjusted outcomes in sensitivity analysis using the Callaway/Sant'Anna staggered difference-in-difference method among patients receiving MV ≥96 hours. Average treatment effect is graphed by time since exposure (year 0 = LTCH closure year); the point estimate and 95% confidence interval at 1 year post-LTCH closure are shown on each graph. Red markers = years prior to LTCH closure; Black marker = year of LTCH closure; Green markers = years after LTCH closure.

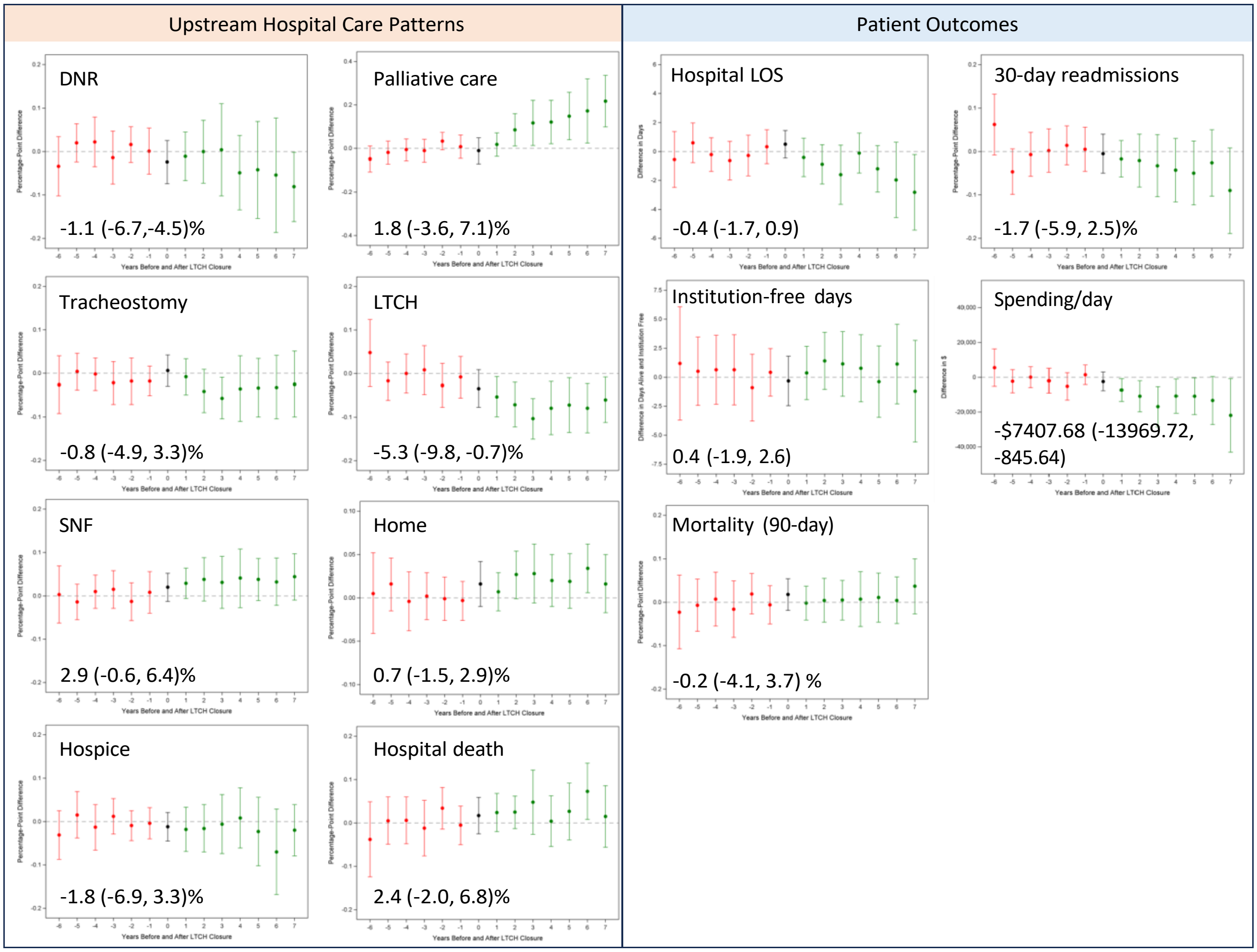

© 2023 Law AC et al. *JAMA Network Open.*

Figure S6. Adjusted outcomes in sensitivity analysis using the Callaway/Sant'Anna staggered difference-in-difference method among patients receiving a tracheostomy. Average treatment effect is graphed by time since exposure (year 0 = LTCH closure year); the point estimate and 95% confidence interval at 1 year post-LTCH closure are shown on each graph. Red markers = years prior to LTCH closure; Black marker = year of LTCH closure; Green markers = years after LTCH closure.

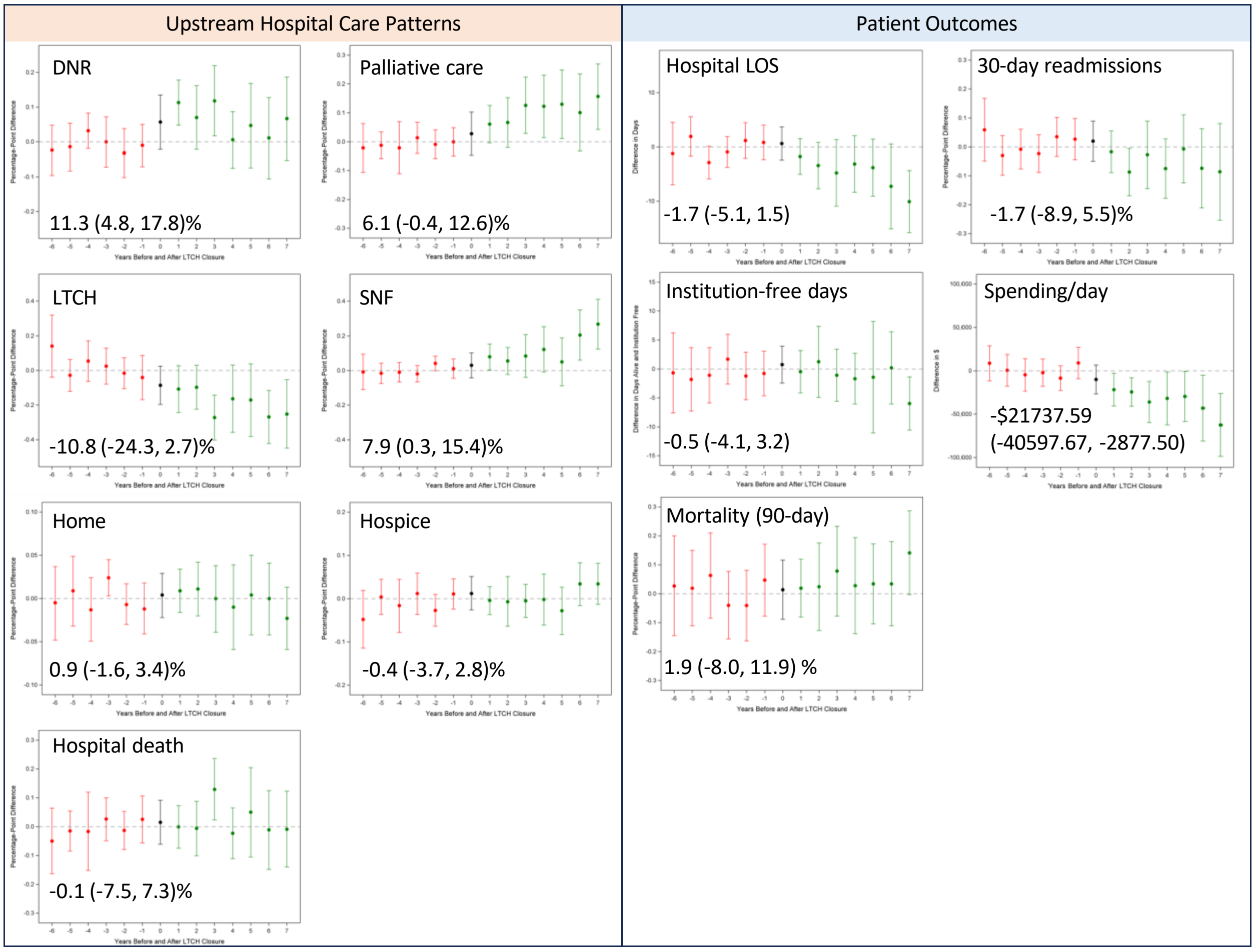

© 2023 Law AC et al. *JAMA Network Open.*
