# Supplementary material for: Patient Outcomes After Long-Term Acute Care Hospital Closures
Source: JAMA Netw Open. 2023 Nov 21;6(11):e2344377. doi: 10.1001/jamanetworkopen.2023.44377 (PMC10663966; doi:10.1001/jamanetworkopen.2023.44377)
Supplement: Supplement 2. — Data Sharing Statement [file jamanetwopen-e2344377-s002.pdf]

Data Sharing Statement

Law. Patient Outcomes After Long-Term Acute Care Hospital Closures. *JAMA Netw Open*.
Published November 21, 2023. doi:10.1001/jamanetworkopen.2023.44377

Data
Data available: No

Additional Information
Explanation for why data not available: Medicare DUA prohibits sharing
